# Harmonizing hope: navigating the osteoarthritis melody through the CCL2/CCR2 axis for innovative therapeutic avenues

**DOI:** 10.3389/fimmu.2024.1387651

**Published:** 2024-07-15

**Authors:** Mitra Abbasifard, Hossein Khorramdelazad

**Affiliations:** ^1^ Department of Internal Medicine, School of Medicine, Ali-Ibn Abi-Talib Hospital, Rafsanjan University of Medical Sciences, Rafsanjan, Iran; ^2^ Department of Immunology, School of Medicine, Rafsanjan University of Medical Sciences, Rafsanjan, Iran

**Keywords:** CCL2, CCR2, osteoarthritis, autoimmune disease, inflammation

## Abstract

Osteoarthritis (OA) is characterized by a complex interplay of molecular signals orchestrated by the CCL2/CCR2 axis. The pathogenesis of OA has been revealed to be influenced by a multifaceted effect of CCL2/CCR2 signaling on inflammation, cartilage degradation, and joint homeostasis. The CCL2/CCR2 axis promotes immune cell recruitment and tips the balance toward degeneration by influencing chondrocyte behavior. Insights into these intricate pathways will offer novel therapeutic approaches, paving the way for targeted interventions that may redefine OA management in the future. This review article explores the molecular symphony through the lens of the CCL2/CCR2 axis, providing a harmonious blend of current knowledge and future directions on OA treatment. Furthermore, in this study, through a meticulous review of recent research, the key players and molecular mechanisms that amplify the catabolic cascade within the joint microenvironment are identified, and therapeutic approaches to targeting the CCL2/CCR axis are discussed.

## 1 Introduction

Osteoarthritis (OA) is characterized by the progression of articular cartilage degradation, inflammation, and changes in joint homeostasis that make osteoarthritis a significant global health burden (1). OA is associated chiefly with weight-bearing joints like the knees, hips, and spine, but it can also affect hands (2). It is associated with symptoms such as joint pain, swelling, and stiffness, with several risk factors including aging, obesity, injuries to joints, genetic predispositions, and certain metabolic conditions (3). Treatment strategies involve symptom management, lifestyle modifications, physical therapy, and, in severe cases, surgical interventions based on medical histories and physical examinations (4–6). Evidence revealed that the immune system and its components play a pivotal role in the pathogenesis of OA because immune cells are activated in OA, contributing to cartilage degradation and joint inflammation (7, 8). Inflammatory mediators are released in OA, contributing to cartilage degradation and joint inflammation, previously considered a non-inflammatory disease (9). A complex interplay of molecular signals is orchestrated by the chemokine (C-C motif) ligand 2/chemokine (C-C motif) receptor 2 (CCL2/CCR2) axis in the pathogenesis of OA (10). As part of the intricate symphony of OA progression, this multifaceted signaling pathway profoundly impacts inflammation, cartilage degradation, and joint homeostasis (11). Inflammation cascades in the joint microenvironment are perpetuated by the CCL2/CCR2 axis because it promotes immune cell recruitment (12). As a result of this orchestrated immune response, the balance tips towards degeneration, influencing the behavior of chondrocytes and exacerbating the joint’s catabolic process (13). OA is multifactorial, involving mechanical stress, genetic factors, and the immune system. A personalized treatment plan emphasizes lifestyle modifications, weight management, and physical therapy to address inflammation and structural changes (14). Moreover, nonsteroidal anti-inflammatory drugs (NSAIDs), corticosteroid injections, and disease-modifying osteoarthritis drugs (DMOADs) can manage the immune response (15). Emerging biological therapies also target specific inflammatory pathways and have promising clinical outcomes in patients with OA (16).

Accordingly, this review offers an in-depth overview of the molecular symphony orchestrated by the CCL2/CCR2 axis in OA, providing valuable insights that could pave the way for innovative and targeted interventions in the future, thereby redefining the management of this prevalent musculoskeletal disorder.

## 2 Understanding osteoarthritis

### 2.1 The onset: tracing the origins of osteoarthritis

Cartilage, which covers the ends of the bones of the joints, gradually breaks down in degenerative joint diseases such as OA (17). As cartilage wears away, bones rub against one another, causing joint pain, stiffness, and reduced mobility (18). As a result of prolonged inactivity or excessive use, OA causes joint pain, swelling, and stiffness. Several risk factors influence the development of OA. As cartilage naturally deteriorates with age, OA may lead to a reduced range of motion, joint instability, and the development of bone spurs over time. Therefore, aging can be another significant risk factor for OA development (5, 19). The development of OA is also associated with obesity since excess weight causes joint stress, with joint injuries and trauma that can accelerate cartilage breakdown (20). Genetic factors, joint overuse, and certain metabolic conditions can cause OA (21). As part of the diagnosis of OA, a physician will assess a patient’s medical history, perform a physical examination, and conduct imaging studies (22). A clinical evaluation aims to determine whether there are characteristic symptoms of joint pain and exclude other possible causes. Symptoms of OA can be managed, and joint function improved through lifestyle modifications, physical therapy, pain medications, and surgical interventions such as joint replacement in severe cases (23). The quality of life of individuals with OA must be improved through early diagnosis and holistic management.

Studies also emphasized the substantial role of trauma in the etiology of OA, particularly focusing on post-traumatic osteoarthritis (PTOA) (24, 25). It highlights that trauma can be responsible for a considerable proportion of OA cases, with U.S. clinical statistics indicating that PTOA represents 12–42% of OA cases (26). Regarding the prevalence and impact of PTOA, noting that knee injuries, mainly those involving the anterior cruciate ligament (ACL) and meniscal damage, considerably increase the risk of developing OA. PTOA has a distinct molecular pathophysiology compared to age-associated OA, which may explain the poor translation of preclinical outcomes to clinical trials. Therefore, there is a requirement for targeted therapeutic interventions and the development of predictive biomarkers to prevent and manage PTOA, emphasizing that understanding trauma-induced OA molecular mechanisms is essential for obtaining promising clinical outcomes (26).

### 2.2 The joint ensemble: cell types and their roles in osteoarthritis progression

Chondrocytes, the cells that reside in cartilage, play a crucial role in maintaining its structural integrity. Every cell in the joint plays a role in the progression of OA (27). The chondrocytes in OA undergo phenotypic changes and produce enzymes that contribute to cartilage destruction, resulting in an imbalance between cartilage degradation and repair (28). Synoviocytes, found in the synovium surrounding joints, become activated in OA, releasing inflammatory cytokines and enzymes that exacerbate joint inflammation and contribute to disease progression (29). Moreover, synovial inflammation promotes the production of joint fluid, which causes swelling and pain in the joint (30). Osteoblasts and osteoclasts play an imperative role in bone remodeling (31). Osteophytes and bone spurs are often present in OA due to excessive bone formation (32). Multiple studies in both experimental models and human OA have revealed an elevation in subchondral bone turnover, including bone resorption and formation, during the initiation and progression of the disease (33–36). Typically, bone resorption and formation are coupled processes, but in certain conditions, such as postmenopausal osteoporosis, they can become imbalanced. This imbalance is also detected in patients with osteoporosis or progressive OA, emphasizing a potential uncoupling of these processes in OA. Expressly, in human hip OA, increased bone resorption and formation have been noted in the subchondral bone beneath mild, moderate, and severe cartilage lesions (37). However, an increase in bone volume was only observed beneath moderate and severe lesions. These observations indicate an increase in the frequency of osteoclast in areas with subchondral osteosclerosis in advanced knee OA. Moreover, recent experimental OA models have further reported this uncoupled bone remodeling, disclosing that altered mechanical loading triggers subchondral bone resorption, followed by enhanced recruitment of osteoprogenitors and formation of osteoid islets in marrow spaces (35, 38, 39). Given the uncoupled nature of bone remodeling in OA, inhibiting bone resorption may benefit both the early (osteopenia) and advanced (osteosclerosis) stages of OA (40, 41). Several types of immune cells, such as macrophages and T lymphocytes, contribute to joint inflammation (42). While traditionally associated with autoimmune responses, T lymphocytes are increasingly recognized for their involvement in the inflammatory processes of OA. Macrophages release pro-inflammatory mediators and enzymes, further accelerating cartilage degradation (43). It is also known that OA also affects the capsule of the joint as well as ligaments and tendons, which are comprised of fibroblasts (44). Fibroblasts produce extracellular matrix (ECM) proteins, and their dysregulation may impact joint stability and function (45). In addition, it has been shown that a non-immune cell, fibroblast-like synoviocytes (FLSs), reside in the synovium’s inner layer. They are specialized mesenchymal cells that play an essential role in collagen homeostasis of the articular joint, provide ECM materials to cartilage, and contribute to joint destruction through multiple mechanisms (46). The intricate interplay between these types of cells is crucial for understanding how OA progresses. The current research focuses on identifying critical molecular pathways and therapeutic targets within this joint ensemble. In order to provide more effective treatments for individuals affected by OA, researchers intend to develop targeted interventions that alleviate symptoms and modify the disease course by elucidating the role of these cells in OA pathophysiology.

### 2.3 Immune system and osteoarthritis

Traditional views of OA as a non-inflammatory form of arthritis have been replaced with recognizing that the immune system plays an essential role in its pathogenesis (47). Immune responses contribute to the inflammation and tissue destruction observed in affected joints, even though OA is primarily characterized by cartilage breakdown and bone changes. OA is caused by the activation of immune cells, which release enzymes and cytokines that contribute to cartilage degradation and inflammation of joints (48). Moreover, OA is characterized by low-grade chronic inflammation in the synovium, which contributes to pain and joint damage and perpetuates the progression of the disease (49). Based on available knowledge, in autoimmune diseases, a wide range of cell types, such as macrophages, neutrophils, NK cells, dendritic cells (DCs), and lymphocytes, are involved in innate and adaptive immune responses (50). Activation of the innate immune system is started upon the ligation of pattern recognition receptors (PRRs) to damage-associated molecular patterns (DAMPs) and pathogen-associated molecular patterns (PAMPs). Toll-like receptors (TLR), RIG-1-like receptors (RLR), and NOD-like receptors (NLR) are prominent immune sensors involved in this process, stimulating nuclear factor kappa B (NF-κB) and mitogen-activated protein kinase (MAPK) pathways, upregulating the expression of interleukin-1β (IL-1β) and tumor necrosis factor-alpha (TNF-α), and inducing cartilage catabolism (51–53). It has been widely reported that TLR-2 and TLR-4 are upregulated in OA lesions compared to unaffected OA cartilage and normal cartilage. Additionally, endogenous signals like hyaluronan and fibronectin may activate TLRs 2 and 4 in OA (54). As another essential component of the innate immune system, the complement system, which consists of components C1-C9 and cofactors, is found in an increased abundance of synovial fluid from OA patients. Inflammatory molecules and membrane attack complex (MAC) formation are affected by genetic deficiencies in complement components C5, C6, and CD59a, indicating that complement plays a pivotal role in the progression of OA (55). In OA, cartilage degradation exposes PRRs, leading to persistent innate immune system activation (48).

It has been revealed that the key components of the adaptive immune system are T cells, divided into cytotoxic T cells (CTLs) and T helper (Th) cells. Th cells are categorized into Th1, Th2, Th9, Th17, and Th22 cells (56). Furthermore, B cells are crucial in developing an adaptive immune response through antibody production (humoral immunity). Still, the T cells play an important role in maximizing the protection against intracellular pathogens and tumors (cellular immunity) (57). Depending on the secretion of cytokines, Th2 cells may influence macrophage polarization toward regenerative M2 or inflammatory M1 phenotypes (58). Conventionally, Th cells are involved in microbe elimination; however, these lymphocytes can also participate in inflammatory phenomena and tissue repair processes by inducing macrophage differentiation in response to tissue-derived mediators in an IL-4-dependent manner (59). There is an emerging area of research into the role of T cells in OA progression (48). Evidence reveals that patients with OA have higher T and B cells than healthy controls (60). In OA patients, synovial fluid and tissue have elevated levels of Th1, Th9, Th17 cells, and CTLs, releasing catabolic cytokines like IL-2, IFN-γ, and TNF-α (61). Further research is needed to understand the specific role of T cells in osteoarthritis progression, even though they are present and contribute to cartilage matrix destruction.

### 2.4 Role of pathogens in osteoarthritis

Pathogens and infections may play a role in OA development via mechanisms involving chronic low-grade inflammation and immune system activation. Evidence revealed that innate immune system-mediated inflammation in OA can be exacerbated by microbiota imbalances and synovial infections, activating toll-like receptors (TLRs) and the complement system, resulting in the degradation of cartilage and synovitis (62, 63). Moreover, gut microbiota can influence OA progression by inducing systemic inflammation, as shown in pre-clinical studies where microbiota isolated from metabolically compromised mice accelerated OA in these animals (64). Furthermore, specific microbes in the joint synovia have been accompanied by hyperinflammation, contributing to OA pathogenesis (65). As discussed, TLRs are a critical component of the innate immune system and play a remarkable role in the pathogenesis of OA. These receptors recognize PAMPs and DAMPs, triggering inflammatory responses. Specifically, TLRs such as TLR2 and TLR4 have been involved in OA via mediating the expression of chemokines like CCL2 (66). Following infections and activation by PAMPs, TLRs trigger signaling pathways that produce inflammatory mediators, including CCL2. Regarding the role of CCL2 in recruiting monocytes and macrophages to sites of inflammation, its increased levels have been detected in the synovial fluid of OA patients. The CCL2/CCR2 axis is critical in recruiting monocytes/macrophages to the OA joint, resulting in synovitis and cartilage destruction. Accordingly, blocking this pathway considerably reduced inflammation and tissue damage in experimental OA models (67). On the other hand, TLR activation by microbial products or endogenous ligands produces pro-inflammatory mediators and MMPs, degrading the cartilage matrix and provoking inflammation. Studies have reported that TLR2 and TLR4 activation in chondrocytes and synoviocytes upregulates the expression of MMPs, further contributing to joint degradation (68, 69).

Increased levels of lipopolysaccharides (LPS), as an important TLR4 ligand, can occur following impaired hepatic clearance, high dietary fat intake, dysregulation of the endocannabinoid system, decreased intestinal motility, and reduced physical activity (70). Beyond LPS, additional metabolites produced by gut microbiota, capable of promoting OA, might penetrate the circulation via a compromised intestinal barrier. Notably, recent studies have identified microbial DNA, including that from common gut microorganisms, in osteoarthritic cartilage and synovial tissue (71, 72). This evidence suggests that increased intestinal permeability may facilitate the translocation of gut microbes to joint tissues (73).

Collectively, activation of the TLRs and the subsequent expression of CCL2 following infections can play a crucial role in mediating the inflammatory response in OA, leading to synovitis, cartilage destruction, and disease progression. Targeting these pathways holds the potential for developing therapeutic approaches to reduce inflammation and tissue damage in OA.

### 2.5 Treatment of osteoarthritis

NSAIDs are commonly used to reduce pain and inflammation associated with OA to alleviate symptoms and slow down its progression (74). A disease-modifying osteoarthritis drug (DMOAD) is a treatment for osteoarthritis aimed at altering the underlying disease process and may have immunomodulatory effects on the immune system (75). Additionally, corticosteroid administration can provide targeted relief in OA (76). The interest in biological therapies that target specific inflammatory pathways involved in OA has increased in recent years (77–79). Specific cytokines, chemokines, or other immune mediators often trigger joint inflammatory responses. Several anti-inflammatory drugs and inhibitors, such as monoclonal antibodies and small molecules, can target and block these ligands or their receptors to reduce inflammation (80–82). OA is a multifactorial disease, and several other factors, such as mechanical stress and genetic predisposition, contribute to its pathogenesis (83). Even though the immune system is known to play a role, several other potential therapeutic targets are involved in the pathogenesis of OA. Various approaches are often used in customized treatment plans to address joint inflammation and structural alterations in OA.

## 3 CCL2 and CCR2: the protagonists

Chemokines and their corresponding receptors are essential in the intricate dance of immune responses. The inflammation and immune regulation narrative is intricately shaped by CCL2 and its receptor CCR2, two key orchestrators among these players.

### 3.1 CCL2: master conductor of inflammation

In the symphony of immune responses, CCL2 is the master conductor, also termed monocyte chemoattractant protein-1 (MCP-1) (84). The gene coding for CCL2, a 13 kDa glycoprotein consisting of 76 amino acids, is located on chromosome 17 at position q11.2 (85). Among the G protein-coupled receptors (GPCR) family members, CCL2 is a potent chemoattractant primarily for monocytes, NK cells, and memory T cells (86). Monocytes, astrocytic cells, endothelial cells, fibroblasts, keratinocytes, mesangial cells, smooth muscle cells, microglia, and neurons can release CCL2 (87, 88). Following the ligation of CCL2 to CCR2, immune cells infiltrate inflammation sites due to these molecules’ ability to recruit and migrate monocytes, T lymphocytes, and DCs (89). Initiating and perpetuating inflammatory cascades depend on its indispensable role in coordinating immune cell trafficking. CCL2 levels are elevated in various pathological conditions, including atherosclerosis, rheumatoid arthritis (RA), OA, diabetes, psoriasis, cancer, and infectious diseases, highlighting its importance in disease pathogenesis (90–94).

Inflammatory stimuli such as IL-1β and TNF-α, as well as microbial products (e.g., Lipopolysaccharide), can increase the expression of CCL2 via various signaling pathways (95, 96). Key pathways include the nuclear factor kappa B (NF-κB) and MAPK pathways, activating transcription factors that bind to the CCL2 promoter, enhancing transcription (97). Extracellular matrix protein cellular communication network factor 1 (CCN1) or Cyr61 has been recognized as a remarkable regulator of CCL2 expression (98). CCN1 can interact with integrins and heparan sulfate proteoglycans to activate NF-κB, increasing CCL2 production (99). This CCN1-related mechanism is critical, as targeting CCN1 could suggest a potential therapeutic tactic for modulating CCL2 levels and subsequent inflammatory responses (100). The induction of CCL2 expression in response to inflammatory stimuli further emphasizes its dynamic role in amplifying immune responses. Upon arrival on the battlefield, this multifaceted chemokine modulates the functions of immune effectors by attracting them. CCL2 expression is regulated by transcriptional, post-transcriptional, and post-translational mechanisms, as shown by studies (101, 102). Understanding CCL2’s role in orchestrating immune responses and its potential as a therapeutic target for various inflammatory conditions requires a deep understanding of its regulatory intricacies.

### 3.2 CCR2 takes the spotlight: its impact on cellular migration and activation

CCR2 plays a crucial role in cellular migration and activation as the designated receptor for CCL2 (103). The CCR2 amino acid sequence exhibits a high degree of identity between species and is a member of the GPCR superfamily (104). Despite sharing conserved cysteine residues with other members of the CCR family, CCR2 has minimal homology with them. CCR2 comprises seven transmembrane domains containing a putative N‐linked glycosylation at amino acid 14, a sulfotyrosine at amino acid 26, a phosphotyrosine by Janus kinase 2 (JAK2) at amino acid residue 139, and a disulfide bond at amino acid 113 ↔ 190. Human CCR2 contains multiple domains, including α-helixes and β-strands (86). CCR2 expression on the surface of monocytes, macrophages, and specific subsets of T lymphocytes acts as a molecular compass, directing immune cells toward the inflammatory sites with high concentrations of CCL2 (105). Cell migration toward the site of injury or infection is facilitated when CCR2 triggers intracellular signaling cascades upon engagement with CCL2 (84).

Activation of CCR2 has been found to modulate immune cell function beyond its canonical role in chemotaxis. The protein plays a role in regulating macrophage polarization, which affects the balance between pro-inflammatory M1 and anti-inflammatory M2 phenotypes. It has been revealed that the CCL2/CCR2 axis is involved in determining the extent of macrophage polarization because CCL2 enhances the LPS-induced production of IL-10 as an anti-inflammatory and inducer of M2 macrophage polarization. At the same time, CCL2 blockade upregulates the expression of M1 polarization-associated genes and cytokines and downregulates the expression of M2-associated markers in human macrophages (106). In addition, *Ccr2^-^* deficient bone marrow-derived murine macrophages demonstrated an M1-skewed polarization profile at the transcriptomic level and significantly increased proinflammatory cytokine expression when LPS was applied. As a result, the CCL2/CCR2 axis influences macrophage polarization by regulating genes relevant to polarization and proinflammatory cytokines (106). According to the available studies, infiltrating immune cells in an inflammatory milieu is associated with overexpression of CCL2 and CCR2. It has been reported that CCR2-driven activation of NF-κB is involved in regulating DC and Langerhans cell maturation (107). In addition, evidence indicates that CCR2-mediated signaling may modulate T-cell differentiation and activation, influencing adaptive immune responses. Studies conducted *in vitro* and *in vivo* have demonstrated that CCR2 directly influences the production of inflammatory cytokines in T cells. In the absence of CCR2 in T cells, the progression of colitis slows down. Additionally, the lack of CCR2 in T cells triggers a process that increases the number of Foxp3^+^ regulatory T cells (Tregs) and reduces the levels of Th17 cells in living organisms. This suggests that CCR2 is crucial in regulating the immune response by balancing the ratio of effector T cells to Tregs (108). Moreover, disorders characterized by dysregulated inflammation may benefit from therapeutic intervention through the intricate regulatory networks governing their expression and activation (109). Therefore, the collaboration between CCL2 and CCR2 in orchestrating immune responses tells an exciting story of molecular interactions influencing inflammation’s course. Understanding the nuances of their roles could lead to the development of targeted therapeutic strategies aimed at fine-tuning immune responses in different pathological conditions.

While the review’s main focus is the CCL2/CCR2 axis, it is advantageous to briefly mention other pivotal pathways responsible for OA pathogenesis, influencing chondrocytes and other cell types. Depending on the context, the Wnt/β-catenin signaling pathway has been revealed to play a dual role, both provoking and suppressing chondrocyte activity (110). NF-κB signaling is another essential pathway that mediates inflammation and catabolic responses, including upregulating MMPs and inducible iNOS (54). The TGF-β pathway, as a double-edged sword, can either stimulate or inhibit cartilage degradation based on the specific receptors and involved downstream signaling (111). Furthermore, AMP-activated protein kinase (AMPK) signaling has been recognized to regulate energy homeostasis and inflammatory responses in chondrocytes (112). The role of fibroblast growth factor 2 (FGF2) signaling in the pathogenesis of OA via modulation of Wnt/β-catenin and other signaling pathways emphasizes its potential therapeutic relevance (113). Hypoxia-inducible factors (HIFs) contribute to chondrocyte survival under low oxygen states typical in OA joints (111). These signaling pathways collectively play expressive roles in OA pathogenesis and are imperative considerations alongside the CCL2/CCR2 axis.

## 4 The intricate dance: CCL2/CCR2 axis in osteoarthritis

**Figure 1:**
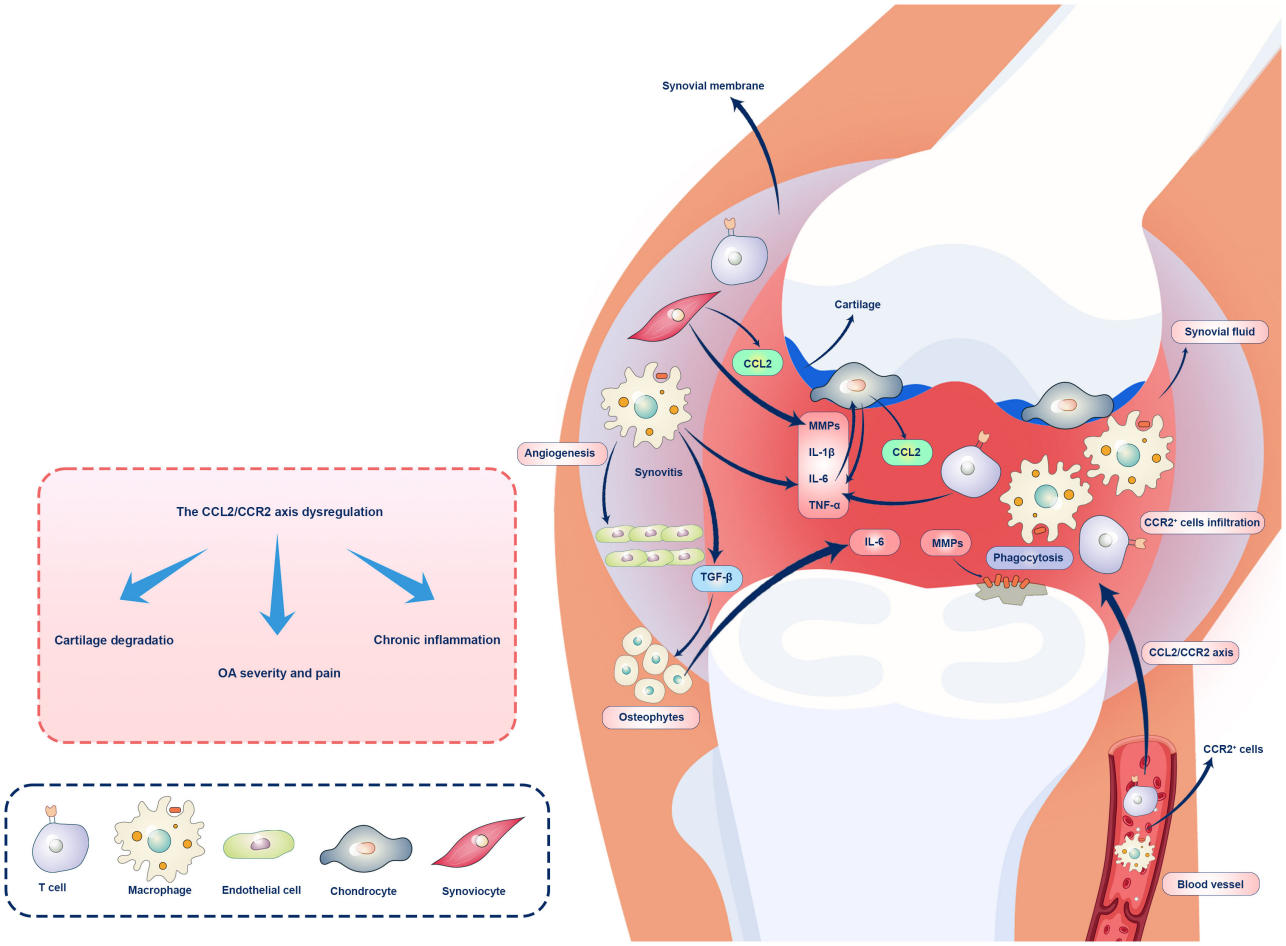
Role of the CCL2/CCR2 axis OA immunopathogenesis. Overexpression of CCL2 in chondrocytes and synovial cells is associated with CCR2^+^ macrophages and T cell recruitments and infiltration in the synovium, chronic inflammation, and OA. CCL2/CCR2 axis regulates inflammation in OA and other pathological conditions, and activation of synovial fibroblasts by pro-inflammatory cytokines leads to CCL2 release and recruitment of CCR2^+^ monocytes and macrophages. Macrophages play a role in preserving inflammation and inducing cartilage damage via cytokine production (IL-1β, IL-6, TNF-α) and MMP activation. Chondrocytes upregulate CCL2 expression in response to mechanical stress and inflammatory signals, leading to cartilage degeneration. CCR2 facilitates cartilage and bone damage independently of synovial macrophage infiltration. Ultimately, the dysregulation of the CCL2/CCR2 axis can lead to joint inflammation and tissue damage.

The CCL2/CCR2 axis is a pivotal player in the inflammatory complex orchestration. This section aims to provide a comprehensive overview of the current knowledge about the CCL2/CCR2 axis in OA, emphasizing the cellular choreography accompanying inflammation and the delicate balance that governs harmony and discord in this dance (**Figure 1**).

### 4.1 Cellular choreography: how CCL2/CCR2 axis orchestrates inflammation

The expression of CCL2 in chondrocytes, osteoblasts, and synovial cells is associated with bone metabolism and OA. In the synovium of patients with OA, CCR2^+^ cells are prevalent (114). The CCL2/CCR2 axis has been demonstrated to regulate inflammation in OA and other pathological conditions (115). Some previous studies showed that this axis orchestrates an intricate cellular choreography during OA pathogenesis (66, 116, 117). In OA, the dynamic interplay between CCL2 and CCR2 is mediated by synovial tissues, chondrocytes, and infiltrating immune system cells. In the synovium, pro-inflammatory cytokines such as IL-1β and TNF-α are responsible for activating synovial fibroblast, releasing CCL2, and recruiting CCR2^+^ monocytes and macrophages (118). Infiltrating macrophages contributes to developing a pro-inflammatory milieu and sustaining inflammation within the joint (119). In addition to their role in innate immunity and tissue remodeling, macrophages produce cytokines and activate matrix metalloproteinases (MMPs), damaging cartilage. Depleting macrophages in mouse models of OA reduced cartilage degeneration and osteophytosis, demonstrating their role in pathologic bone formation (120). A study reported that S100A8 and S100A9 proteins are mainly produced by GM-CSF–differentiated macrophages in the synovium, with lesser production by M-CSF–differentiated macrophages and not by fibroblasts (121). S100A9 stimulation of OA synovial tissue led to increased production of IL-1β, IL-6, IL-8, and TNF-α, as well as upregulation of MMPs. Further experiments revealed that these effects were mainly due to the stimulation of GM-CSF–differentiated macrophages and, to a lesser extent, M-CSF-differentiated macrophages, while fibroblasts remained unaffected by S100A9. Additionally, S100A9 activated canonical Wnt signaling in GM-CSF-differentiated macrophages but not M-CSF-differentiated ones. Using the S100A9 inhibitor paquinimod on OA synovium reduced the activation of this signaling pathway (121, 122). CCL2/CCR2 dance is also actively performed by chondrocytes, the primary cells of articular cartilage. The expression of CCL2 is increased in chondrocytes in response to mechanical stress and inflammatory signals, resulting in a positive feedback loop that promotes inflammation and cartilage degeneration (123). This process is further exacerbated by the infiltration of CCR2^+^ immune cells into the cartilage, resulting in a progressive loss of cartilage integrity (66).

However, an investigation explored the role of CCR2 in the pathogenesis of injury-induced OA. Utilizing a murine model of injury-induced OA through DMM, CCR2 was systemically blocked using the antagonist RS504393 at various stages of disease progression. Joint degeneration was assessed by evaluating cartilage (including metrics such as cartilage loss, chondrocyte hypertrophy, and MMP-13 expression) and bone lesions (such as bone sclerosis and osteophyte formation) with and without CCR2 inhibition. Pain-related behaviors were also studied by measuring weight distribution between normal and arthritic hind paws using the IITS incapacitance meter. This study showed that the timing of CCR2 antagonist administration was crucial for mitigating joint damage. Early intervention with the CCR2 antagonist within the first four weeks post-injury significantly reduced OA-related cartilage and bone damage. In contrast, later interventions proved less effective. Furthermore, early to moderate CCR2 blockade (1–4 weeks and 4–8 weeks post-surgery, respectively) resulted in decreased pain measures, with sustained pain improvement even after cessation of treatment. These results indicate the potential of early CCR2 antagonism to not only decelerate the progression of post-injury OA but also to alleviate associated pain symptoms, suggesting a therapeutic window for optimizing the efficacy of CCR2-targeted treatments in OA management (124).

It has been demonstrated that CCL2 treatment activated ERK and p38 via CCR2 in healthy chondrocytes. CCL2 treatment of healthy chondrocytes for short (6h) or long (7–72h) periods resulted in upregulating *CCR2, MMP-1, MMP-3, MMP-13*, and tissue inhibitor of matrix metalloproteinase 1 (*TIMP1*). CCL2-mediated upregulation of CCR2 and cartilage catabolic markers was mediated by ERK and p38 signaling in healthy and OA chondrocytes (125). Further, the CCL2/CCR2 axis influences how different types of cells interact in the joint microenvironment. CCR2^+^ osteoclast precursors are recruited by CCL2, resulting in bone resorption and the characteristic subchondral changes observed in patients with OA (126).

A study explored whether the specific inactivation of the *Ccr2* gene in osteoblasts at various stages of PTOA can improve joint structure, bone parameters, and pain outcomes (126). This study used a tamoxifen-inducible system to inactivate *Ccr2* in collagen1α-expressing cells, resulting in osteoblasts deficient in *Ccr2* (CCR2-Col1αKO). The PTOA model was induced in both CCR2-Col1αKO and control (*CCR2^+/+^*) mice using the medial meniscus (DMM) method destabilization. Recombination was triggered before or after the DMM procedure to compare early versus late inactivation effects. Joint damage was assessed at two, four, eight, and twelve weeks post-DMM using various metrics, including articular cartilage structure (ACS), Safranin-O staining, histomorphometry, osteophyte size and maturity, subchondral bone thickness, and synovial hyperplasia. Pain levels, both spontaneous and evoked, were monitored for up to 20 weeks. Results demonstrated that early inactivation of *Ccr2* in osteoblasts delayed the progression of articular cartilage damage and matrix degradation compared to *CCR2^+/+^* controls, and it also decreased DMM-induced bone thickening. The formation and maturation of osteophytes were only slightly influenced. Conversely, late inactivation of collagen1α-*Ccr2* resulted in less pronounced improvements. Furthermore, *Ccr2* deletion in osteoblasts improved static pain measures, though evoked pain remained unchanged. This study demonstrates that *Ccr2* expression in osteoblasts plays a critical role in the progression of PTOA and associated pain by impacting both cartilage and bone tissues (126).

Taken together, due to the intricate choreography orchestrated by the CCL2/CCR2 axis, OA progression is a multifaceted phenomenon.

### 4.2 Harmony or discord: balance and imbalance in the CCL2/CCR2 dance

Dysregulation of the CCL2/CCR2 dance can lead to pathology, crucial to maintaining a delicate balance. It has been suggested that disruptions in the equilibrium between CCL2 and CCR2 are responsible for the severity and progression of OA (127). A CCL2/CCR2 axis-mediated mechanism is thought to mediate the pain of OA, suggesting that increased CCL2 levels during OA may indicate altered bone and cartilage metabolism, causing pain and destruction of joints (114). There is evidence that elevated CCL2 levels in synovial fluid and serum are related to OA severity and pain (128, 129). As a result of dysregulated CCL2 production in response to inflammatory stimuli, chronic inflammation is perpetuated, leading to relentless cartilage degradation. A positive correlation has been reported between synovial fluid CCL2 concentrations, the Western Ontario and McMaster Universities Arthritis Index (WOMAC) pain ratings, WOMAC function scores, and total WOMAC scores (128). This association remained considerable even after adjusting for potential confounding factors through multivariate linear regression analysis. Additionally, the correlation between synovial fluid and serum CCL2 concentrations with the K-L grading system has been evaluated, and no significant difference was found, suggesting that CCL2 concentrations associated with radiographic changes in knee OA patients were not linked to symptomatic severity (128). These findings suggest that CCL2 synovial fluid levels could be a novel biochemical marker reflecting pain severity and functioning as a pain mediator in knee OA patients. These outcomes may pave the way for new, targeted therapeutic approaches to alleviate pain and its associated symptoms in patients with knee OA.

Excessive activation of inflammatory pathways due to dysregulated CCR2 signaling can lead to joint inflammation and tissue damage. In contrast, inadequate activation of CCR2 may result in a lack of immune cells recruited to repair tissues and resolve inflammation (130). Furthermore, the interplay between the CCL2/CCR2 axis and other signaling pathways adds another layer of complexity to the dance. Cross-talk with Transforming Growth Factor-beta (TGF-β), NF-κB, and MAPK pathways modulate the inflammatory response in OA (131). Consequently, Disease progression can be positively or negatively affected depending on how these interactions are balanced.

A potential role for TGFα-EGFR signaling in OA progression has been identified, particularly in its untested role *in vivo* (132). Except for endothelin A, the downstream effectors of TGFα signaling in cartilage are poorly understood. It has been shown that EGFR signaling induces the expression of CCL2 in osteoblasts, and CCL2 is elevated in human OA cartilage, which promotes proteoglycan loss (127). When CCL2 is elevated, its expression is further driven by a positive feedback loop that prevents mesenchymal progenitor cells from differentiating along chondrogenic lines, inhibiting natural joint repair processes (133). It was revealed that exposure to CCL2 prompts synovial multipotent progenitor cells (sMPCs) pro-inflammatory activation (increasing the expression of IL-6 in sMPCs) while impeding their ability to undergo chondrogenesis *in vitro*, the process by which these cells mature into cartilage. Furthermore, exposure to CCL2 at levels typically found in OA knee joint synovial fluid induces significant alterations in the gene expression profile of sMPCs. Additionally, prolonged exposure to CCL2 results in the heightened expression of CCL2 itself within sMPCs, setting up a positive feedback loop from which these cells struggle to disengage. Consequently, elevated CCL2 can attract sMPCs to the site of injury but also disrupt the normal transcriptional regulation of these cells, hampering their progression toward chondrogenesis (133). These findings present promising avenues for further exploring the mechanisms underlying the observed lack of intrinsic repair following articular cartilage injury and/or arthritis.

The interaction between TGF-α and CCL2 plays a critical role in the pathogenesis of OA (134, 135). A study elucidated that TGF-α stimulates the expression of CCL2 through various intracellular signaling pathways, including MEK/ERK, p38 MAPK, and phosphoinositide 3-kinases (PI3Ks) in chondrocytes (136)​​. TGF-α-induced CCL2 expression subsequently promotes the production of MMPs, specifically MMP-3, which contributes to the degradation of type II collagen and aggrecan, essential components of cartilage extracellular matrix​​. Pharmacologic inhibition of either TGF-α or CCL2 signaling has been revealed to reduce cartilage degradation and disease progression in a rat model of posttraumatic OA, suggesting that the TGF-α/CCL2 axis is a significant pathway in OA pathophysiology (136). These findings indicate that targeting the TGF-α/CCL2 signaling pathway could be a promising therapeutic approach to mitigate cartilage destruction in OA​​.

Polymorphisms, while not directly involved in the pathogenesis of OA, can considerably increase the risk of developing the disease. These genetic variations can impact the biological pathways associated with OA, thereby acting as imperative prognostic biomarkers (137). By identifying individuals with specific polymorphisms, it is possible to predict the probability of OA onset and progression, enabling more personalized approaches to prevention and management. In this context, a study found that specific variations in the CCL2 gene, rs1024611 and rs4586, significantly increased the risk of developing OA, emphasizing the central role played by the CCL2/CCR2 axis in disease susceptibility (138). After further analysis, it was determined that the rs1024611 polymorphism was associated with increased risk for males and non-drinkers. In contrast, the rs4586 polymorphism was associated with heightened risk for smokers and drinkers. As a result of the rs4586 polymorphism, individuals with the CC genotype showed significantly higher levels of CCL2 compared to those with TT genotypes. As a result, variation in CCL2, particularly rs1024611 and rs4586, is associated with OA susceptibility and could serve as early diagnostic markers (138). Another study revealed that certain genetic variations within the *CCL2* gene might be linked to an increased risk of developing knee OA (139). Specifically, the rs2857657 variant (G) allele appears more common in female knee OA cases than in individuals without OA. Additionally, identifying a haplotype (H5) exclusively in the control group implies a potential protective effect against knee OA (139). These findings entail that *CCL2*-associated genetic factors could contribute to the susceptibility to knee OA.

Collectively, CCL2 appears to be a crucial downstream mediator in the cartilage degradation process, including the MEK/ERK pathway, according to the study.

## 5 Clinical implications and therapeutic perspectives

**Figure 2:**
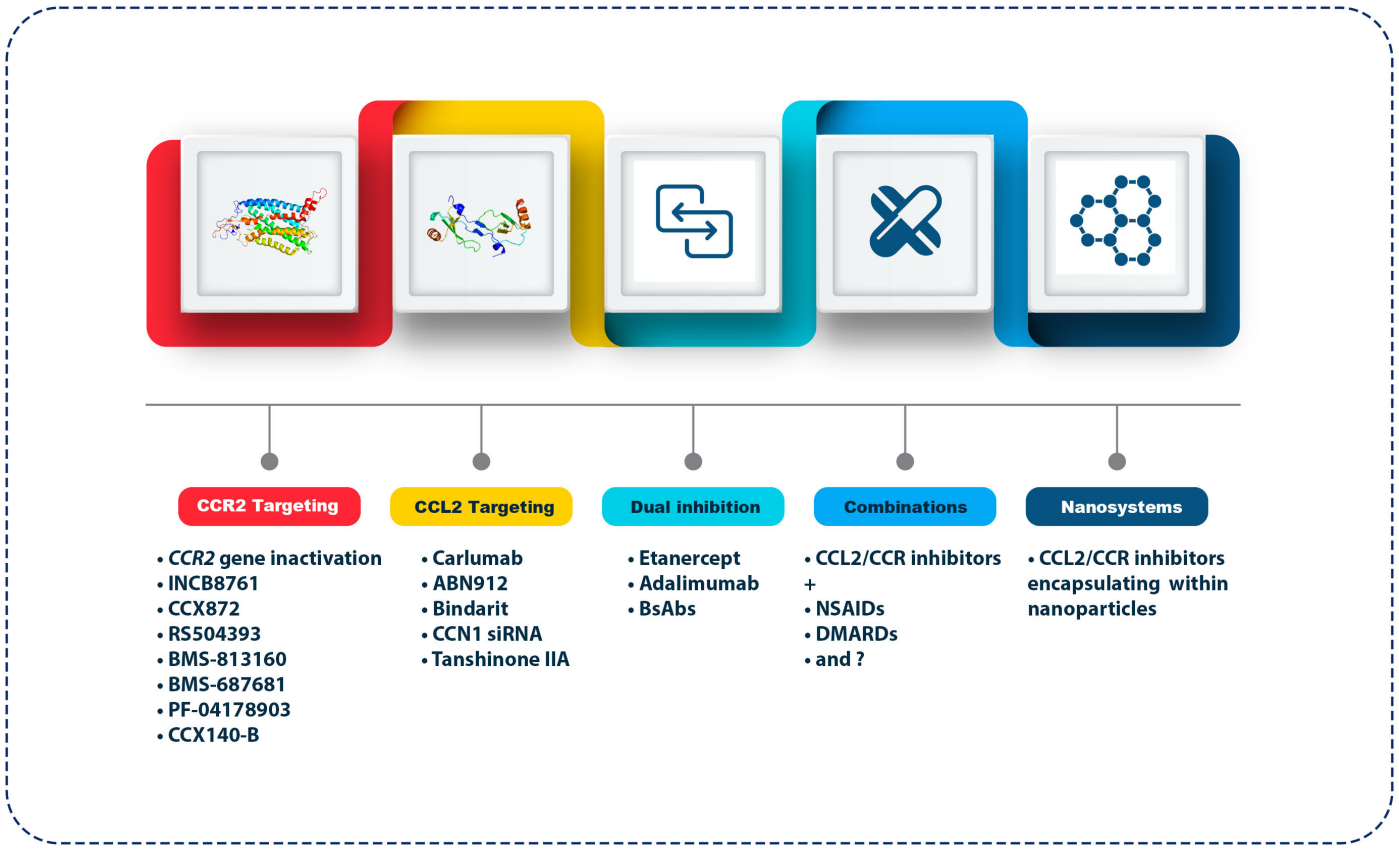
Therapeutic approaches targeting the CCL2/CCR2 axis in OA. The figure outlines various strategies targeting the CCL2/CCR2 axis for OA treatment. CCR2 targeting agents include gene inactivation and antagonists like INCB8761, CCX872, RS504393, BMS-813160, BMS-687681, PF-04178903, and CCX140-B, which block inflammatory cell recruitment. CCL2 targeting includes monoclonal antibodies (Carlumab, ABN912), Bindarit (an NF-κB pathway inhibitor), CCN1 siRNA, and Tanshinone IIA (a traditional medicine extract). Dual inhibition strategies combine CCL2/CCR2 inhibitors with TNF-α inhibitors (Etanercept, Adalimumab) or bi-specific antibodies. Combination therapies pair CCL2/CCR2 inhibitors with NSAIDs or DMARDs. Lastly, nanosystems utilize nanoparticles for targeted delivery of these inhibitors, enhancing bioavailability and reducing systemic side effects.

With the emergence of targeted therapies for various chronic inflammatory diseases, such as RA and OA, focusing on the CCL2/CCR2 axis and its clinical implications can offer new perspectives on how to manage these common and debilitating joint diseases (**Figure 2**). The clinical implications and therapeutic perspectives of these emerging strategies reveal their potential to reshape the treatment landscape for OA. Treatment of OA requires a personalized approach due to the heterogeneity and diversity of the disease (140). By targeting the CCL2/CCR2 axis, interventions can be tailored to patients’ underlying inflammatory profiles. Developing biomarker-driven therapeutic approaches that identify patients with elevated CCL2/CCR2 signaling could pave the way for more effective and personalized treatments (141). Diagnostic tools could be developed to stratify patients by investigating the genetic and molecular signatures associated with the dysregulation of CCL2/CCR2 in OA. Precision medicine facilitates OA management because clinicians can identify individuals more likely to respond positively to CCL2/CCR2-targeted treatments (142, 143). As the current treatment for OA focuses on symptom relief, pain management, and joint function improvements, targeting the CCL2/CCR2 axis may offer a dual benefit of alleviating symptoms and possibly modifying the disease’s course (144, 145). These therapies may alter OA’s natural history by suppressing the inflammatory cascade within the joint. Disease modification can reduce OA progression, offering great hope to patients (146). As a result of this paradigm shift, OA therapeutics have gained significant advancements from purely symptomatic relief to interventions with disease-modifying capabilities. For instance, the role of microRNA-183 (miR-183) in OA pain and its underlying mechanisms was investigated (147). Clinical samples from OA patients and a surgically induced OA pain mouse model were used. Expression levels of miR-183 and various factors linked to OA pain, including TGFα, CCL2, IL-1β, IL-6, TNF-α, transient receptor potential vanilloid subtype-1 (TRPV1), and voltage-gated sodium channels Nav1.3, Nav1.7, and Nav1.8) were measured. The results showed downregulation of miR-183 in both human OA tissue samples and the OA mouse model. Overexpression of miR-183 in mice inhibited the expression of proinflammatory cytokines and pain-related factors in dorsal root ganglia (DRG), alleviating OA pain. Moreover, miR-183 suppressed macrophage infiltration in DRG, directly targeting TGFα (147). These outcomes indicate that miR-183 can ameliorate OA pain by inhibiting the TGFα-CCL2/CCR2 signaling axis, emphasizing its potential as a therapeutic target for OA treatment.

To effectively navigate the complex pathology of OA, CCL2/CCR2-targeted therapies can be combined with other anti-inflammatory agents to achieve synergistic effects. Several signaling pathways are involved in the orchestration of inflammation in OA, and targeting these pathways simultaneously may lead to more profound therapeutic outcomes (148). In addition to nonsteroidal anti-inflammatory drugs (NSAIDs) or disease-modifying anti-rheumatic drugs (DMARDs), combining CCR2 antagonists or anti-CCL2 antibodies with existing therapies could help manage OA in a multifaceted way (149–151). These combination therapies may enhance OA treatment efficacy while minimizing adverse effects, signifying a potential approach to the intricate interplay of inflammatory mediators in OA.

### 5.1 Tuning the orchestra: current and potential therapeutic approaches

#### 5.1.1 CCR2 targeting

A prime target for interventions to regulate immune cell infiltration and inflammation within joints is CCR2, which is predominantly expressed on monocytes and macrophages’ surfaces (152). Therefore, CCR2 targeting by various approaches can be a promising avenue in the search for OA therapeutics. A recent study evaluated sCCR2 E3 gene therapy against OA in an animal model induced by monosodium iodoacetate (MIA). The effects of *sCCR2 E3* gene expression were compared to control groups that received either full-length sCCR2 or an empty vector through intraarticular injection. It has been shown that *sCCR2* gene expression reduces pain severity, suggesting its potential to alleviate pain in OA. Furthermore, micro-computed tomographic analyses revealed a protective effect on bone integrity conferred by sCCR2 E3 activation based on enhanced bone resorption (152). These outcomes were further corroborated by histological assessments demonstrating anti-inflammatory properties and protective effects against cartilage damage in joints and intestines. These findings suggest that *sCCR2 E3* therapy might be an effective treatment for OA, as it inhibits cartilage degradation, alleviates intestinal inflammation, and mitigates pain. Preclinical studies have demonstrated that small molecules that inhibit CCR2 disrupt the chemotaxis of inflammatory cells toward arthritic joints (145).

Another study examined the influence of *Ccr2* inactivation in aggrecan-expressing cells on PTOA progression, joint structures, synovial thickness, and pain (153). Through tamoxifen-inducible *Ccr2* inactivation in skeletally mature mice, the researchers detected that early deletion of *Ccr2* in aggrecan-expressing cells led to substantial improvements in articular cartilage structure, cartilage area and Safranin-O staining compared to mice without *Ccr2* deletion. Moreover, delayed subchondral bone thickness upsurge and delayed osteophyte maturation were noted following early recombination. While late aggrecan-*Ccr2* deletion exhibited some improvement in cartilage health, most data did not reach statistical significance. Remarkably, early *Ccr2* deletion enhanced weight-bearing capacity and mechanosensitivity, specifying improved pain measures compared to control mice (153). These outcomes propose that *Ccr2* inactivation in aggrecan-expressing cells alleviates the initiation, but not the progression, of PTOA, highlighting a potential therapeutic target for managing osteoarthritic pain and joint damage (**Table 1**).

**Table 1:** Therapeutic approach targeting the CCL2/CCR2 axis in OA (Directly and indirectly).

Intervention	Outcome	Ref
**Tamoxifen-inducible *Ccr2* inactivation**	Improvement of articular cartilage structure Enhancement of cartilage area Increase in Safranin-O staining Delay in subchondral bone thickness increase Delay in osteophyte maturation Improvement in weight-bearing capacity Enhancement of mechanosensitivity	(153)
**RS504393 (CCR2 antagonist)**	Reduction in cartilage and bone damage Diminished effectiveness with subsequent treatments Decrease in pain measures Enhancement of pain relief Reduction of cartilage damage, synovitis, and osteophyte formation Reduction of the number of F4/80-positive macrophages	(66, 124)
**Anti-CCN1 siRNA and Tanshinone IIA**	Inhibition of CCN1 reduces senescence markers Inhibition of CCN1 decreases inflammatory markers Inhibition of CCN1 reduces matrix degradation Tanshinone IIA (TanIIA) reduces CCN1 expression in a dose- and time-dependent manner TanIIA treatment decreases senescence and inflammatory markers TanIIA preserves cartilage integrity in human cartilage explants and aged mice Intra-articular injection of TanIIA inhibits CCN1-driven cartilage inflammaging and OA progression in mice TanIIA-treated mice show reduced cartilage damage and lower OARSI scores	(154)
**RS-PLs nanosystem**	Increase in release of CCR2 inhibitor (RS504393) Improvement of early and severe PTOA stages Reduction of bone damage Decrease in synovial hyperplasia Prevention of extracellular matrix loss	(155)

INCB8761/PF-4136309, a potent CCR2 antagonist that blocks the binding of CCL2 to CCR2 receptors on monocytes, myeloid-derived suppressor cells (MDSCs), and macrophages, thereby inhibiting their migration and reducing inflammation with high selectivity, weak hERG activity, high free fraction in protein binding, and a promising *in vitro* and *in vivo* ADMET (ADME and toxicology) profile, was introduced by a study (156). A phase 1b clinical trial showed that following administration of PF-4136309 in patients with pancreatic ductal adenocarcinoma (PDAC), levels of CD14 ^+^ CCR2^+^ inflammatory monocytes reduced in the peripheral blood (157). Regarding the role of CCR2^+^ CD14^+^ inflammatory monocytes in the pathogenesis of OA, it is possible that using PF-4136309 in OA patients would be beneficial by reducing the frequency of these cells at the site of inflammation (158). Fluorouracil (5-FU) is a chemotherapy agent that, in addition to its cytotoxic effects on cancer cells, can induce immunogenic cell death, which enhances the anti-tumor immune response and modulates the TME (159). Moreover, CCX872-B, another CCR2 antagonist, disrupts the CCL2/CCR2 signaling pathway, reducing monocyte recruitment and subsequent tumor-promoting inflammation (160). The combination of FOLFIRINOX (5-FU, leucovorin, irinotecan, oxaliplatin) and CCX872-B showed promising results in patients with locally advanced or metastatic pancreatic cancer (161). The regimen was given to patients for up to 24 weeks, with progression-free survival being the primary endpoint. The secondary endpoints were tumor control and objective response rates. With a tumor control rate of 78% and an ORR of 37% among 50 patients enrolled, 35 completed the 12-week treatment. There was no significant increase in severe adverse events compared to historical data with FOLFIRINOX alone for CCX872-B. For patients with pancreatic cancer, the combination therapy demonstrated encouraging efficacy and acceptable safety profiles, suggesting potential benefits (161). Accordingly, CCX872 is hypothesized to dampen the inflammatory cascade by inhibiting inflammatory monocyte infiltration into the joint by interfering with CCR2 signaling in OA. While these mechanisms have shown promising outcomes in cancer studies, extrapolating them to OA requires caution due to the distinct pathophysiological contexts of cancer and OA. In OA, the primary focus is on chronic inflammation and cartilage degradation, suggesting that while CCR2/CCL2 inhibition could reduce inflammation, the direct effects and safety profiles need thorough investigation in OA-specific models.

Neuropathic pain and OA pain are two distinct forms of chronic pain, each with unique underlying mechanisms. Neuropathic pain arises from damage or dysfunction within the nervous system, either peripheral or central (162). This type of pain is characterized by peripheral sensitization, where injury to peripheral nerves (such as from diabetes, shingles, or trauma) leads to abnormal electrical signals and ectopic activity, causing spontaneous and aberrant nerve firing. Central sensitization also plays a critical role, involving increased excitability and synaptic efficacy in central neurons, often leading to pain amplification and persistent pain (163). In contrast, OA pain is primarily nociceptive, resulting from joint degeneration and the associated inflammatory processes (164). The mechanical wear and tear of cartilage, subchondral bone changes, and synovitis contribute to pain by activating nociceptors (165). Inflammatory mediators released during joint degeneration sensitize these nociceptors, resulting in the characteristic pain of OA (166). While neuropathic pain is primarily driven by neural dysfunction, OA pain is rooted in structural joint changes and inflammation, highlighting the distinct pathophysiological pathways underlying these chronic pain conditions. RS504393 is a specific CCR2 inhibitor that similarly blocks CCL2 binding, diminishing inflammatory cell infiltration and activity (66). To further substantiate the pathogenic role of CCL2/CCR2 signaling in the development of OA in mice was evaluated. It was demonstrated that RS-504393 significantly reduced cartilage damage, synovitis, and osteophyte formation. Additionally, the number of F4/80-positive macrophages was markedly lower in RS-504393-treated mice than those treated with the vehicle (66). These findings suggest that pharmacological inhibition of the CCL2/CCR2 chemokine axis effectively suppresses OA progression in mice, partly by decreasing synovial macrophage accumulation. In another study, RS504393 was administered to OA patients early or delayed to examine the effects on joint damage. According to the results, pharmacological blockade of CCR2 had differing effects on cartilage damage and bone damage based on the timing of administration (124). After the initial 4 weeks following injury, administering the RS504393 significantly reduced cartilage and bone damage associated with OA, but effectiveness decreased with subsequent treatments. In addition, pain-related behavioral studies found that blocking CCR2 signaling during the early (1–4 weeks after surgery) or moderate (4–8 weeks post-surgery) stages of OA led to decreased pain measures, with sustained improvement even after stopping treatment (124). Accordingly, an early intervention targeting CCR2 may slow the progression of post-injury OA and improve pain symptoms, suggesting that CCR2 antagonists could be highly effective in managing OA (**Table 1**). It seems that the mechanism of RS504393 is reducing synovial inflammation, inhibiting joint destruction, and ameliorating clinical symptoms via suppression of the CCL2/CCR2 axis signals (167, 168). In this context, a study used RS504393 to target the MCP-1/CCR2 axis, and the findings showed anti-inflammatory therapeutic potential in animal models of RA (168).

It has been found that changes in the signaling of chemokines and receptors within sensory neurons contribute to the maintenance of neuropathic pain behaviors (169). As a result, molecular mechanisms governing chemokine and receptor interactions in sensory neurons will likely play an essential role in sustaining neuropathic pain after an initial injury or insult (170). These changes may be triggered by various signaling pathways that sensitize sensory neurons, resulting in persisting neuropathic pain. It may be possible to develop novel therapeutic strategies for managing neuropathic pain conditions by understanding and targeting these alterations in chemokine/receptor signaling. It has been reported that (R)-4-Acetyl-1-(4-chloro-2-fluorophenyl)-5-cyclohexyl-3-hydroxy-1,5-dihydro-2H-pyrrol-2-one (CCR2 RA [R]), a CCR2 antagonist, at 14 and 28 days after nerve injury significantly reduced bilateral nociceptive behavior (pain sensitivity). In this phase following nerve injury, the CCL2/CCR2 axis is essential in maintaining pain hypersensitivity (171). According to these findings, it is possible that using this CCR2 antagonist reduces pain in patients with OA. In addition to the mentioned CCR2 inhibitors, BMS-813160 (172), BMS-687681 (173), PF-04178903 (174), and CCX140-B (175), as other potent CCR2 inhibitors used in inflammatory-based disorders, may be beneficial in managing OA.

#### 5.1.2 CCL2 targeting

Another approach to modulating the CCL2/CCR2 axis is targeting the chemokine. A monoclonal antibody (mAb), Carlumab, was previously known as CNTO888)human IgG1κ), which possessed a high affinity and specificity for human CCL2 (176). Carlumab binds to CCL2, hindering its interaction with CCR2 and disrupting the chemotactic signal that drives immune cell recruitment. A preclinical study found systemic carlumab administration repressed prostate tumor growth, reducing CD68^+^ macrophage infiltration and tumor microvascular density following monotherapy (177). Clinical outcomes of a phase 1 trial also revealed that carlumab was well tolerated at 15 mg/kg administered every two weeks in patients with solid tumors (178).

There are intriguing insights into the dynamics of treating cancer patients with Carlumab to target CCL2. After an infusion, carlumab temporarily suppresses free CCL2 levels, but these effects are transient, and levels resume or exceed baseline within a week (179). This contrasts with other mAbs like Omalizumab, where sustained suppression of target levels is observed. The inability of Carlumab to maintain prolonged suppression suggests potential compensatory mechanisms at play, possibly involving increased CCL2 production in response to inhibition. Moreover, the observation of elevated free CCL2 levels, particularly in patients receiving higher doses of Carlumab and associated with worse outcomes, raises questions about the role of CCL2 in disease pathogenesis and the potential overstimulation of compensatory mechanisms. Possible overstimulation of compensatory mechanisms, particularly in patients receiving high doses of Carlumab, is raised by elevated CCL2 levels, particularly in patients receiving higher doses (179, 180). Despite Carlumab’s lack of efficacy in treating progressive idiopathic pulmonary fibrosis (IPF), these findings suggest that newer agents are needed to suppress CCL2-mediated signaling more effectively. This could provide hope for future therapeutic advances in dealing with related diseases (180). There are several vital reasons why clinical trials for ABN912 (another anti-CCL2 mAb) and Carlumab, intended to treat RA and cancer, have been unsatisfactory. Due to the transient neutralization of free-CCL2, a significant accumulation of total-CCL2 in the bloodstream contributed to the limited therapeutic efficacy (176). Pharmacokinetic/pharmacodynamic (PK/PD) modeling emphasized a substantial mismatch between the *in vitro* and *in vivo* binding kinetics, excluding the possibility of treatment-induced amplified CCL2 production as an explanation (179). A significant obstacle posed by CCL2 and Carlumab’s target was the high turnover rate of these proteins (181). As a result, improving bioanalytical tools to measure free ligands *in vivo* was emphasized, as this could provide crucial insights into sustaining target inhibition over time (182). In murine models of metastatic breast cancer, it has been demonstrated that prolonged inhibition of free-CCL2 is essential for the clinical development of any CCL2-targeting agent and can mitigate metastasis formation after treatment cessation (183, 184).

An alternative way to inhibit CCL2 expression is to block the regulator of its expression. As an anti-inflammatory molecule, Bindarit (AF 2838 or 2-methyl-2-[[1-(phenylmethyl)-1Hindazol-3-yl] methoxy] propanoic acid) inhibits CCL2 synthesis and reduces CCL2 production by inhibiting the NF-κB pathway (185, 186). Several preclinical and clinical studies have shown that Bindarit, via suppressing the production of CCL2 and reducing the recruitment of monocytes and macrophages, is effective in treating inflammatory-based disorders, such as diabetes-associated periodontitis (animal model study) (187), lupus nephritis (human study) (188), Alzheimer’s disease (*in vitro* study) (189), and experimental autoimmune encephalomyelitis (EAE) (animal model study) (190). Tanshinone IIA extracts anti-inflammatory properties from the extensively utilized traditional Chinese medicine Danshen (*Salvia miltiorrhiza*) (191). Researchers conducted experiments using isolated primary human chondrocytes, cartilage explants, and a pre-clinical mice model to elucidate how CCN1 contributes to cartilage aging and inflammation (154). They found that CCN1 expression increases with age and is associated with cartilage degeneration. Overexpression of CCN1 promoted chondrocyte senescence and inflammation, while its inhibition via small interfering RNA (siRNA) or the administration of Tanshinone IIA (TanIIA) reduced inflammatory responses and preserved cartilage integrity (154). These findings indicate that targeting CCN1 signaling could be a potential therapeutic strategy for mitigating OA progression by reducing senescence-associated secretory phenotype (SASP) factors and preserving cartilage homeostasis. According to the CCN1 inducing CCL2 through FAK, PI3K/Akt, and NF-κB pathways in retinal vascular endothelial cells (192), inhibiting CCN1 can be beneficial in suppressing CCL2 and reducing the infiltration of monocytes and macrophages in the synovium and related inflammatory responses in OA (100) (**Table 1**).

An investigation revealed that CCN1 also plays a crucial role in RA pathogenesis. CCL2 is the critical chemokine that regulates the migration and infiltration of monocytes in RA. This study reported higher levels of CCN1 and CCL2 in synovial fluid from RA patients compared to non-RA controls. The MAPK signaling pathway mediates the CCN1-induced increase in CCL2 expression, and CCN1 negatively regulated miR-518a-5p expression via the MAPK cascade. In contrast, inhibition of CCN1 expression with lentiviral vectors expressing short hairpin RNA ameliorated articular swelling, cartilage erosion, and infiltration of monocytes in the ankle joints of mice with collagen-induced arthritis. Regarding the similar role of CCL2 in the pathogenesis of RA and OA, CCN1 inhibition could serve as a potential strategy via reducing the CCL2/CCR2 axis-mediated inflammation in these bone and cartilage disorders (98).

#### 5.1.3 Dual inhibition strategies

To achieve a more comprehensive therapeutic effect, researchers are exploring dual inhibition strategies recognizing the intricate crosstalk between different signaling pathways within the joint microenvironment (66). Combining CCL2/CCR2-targeted agents with inhibitors of other inflammatory mediators, such as TNF-α or IL-1β, may offer synergistic benefits (193). An investigation on patients with RA showed that administrating anti-TNF-α antibodies (infliximab) downregulated CCL2 levels, while the concentrations of CCL3 and IL-8 remained unchanged (194). Another study assessed the effects of etanercept and adalimumab (TNF-α inhibitors) on the expression of CCL2 and evaluated potential intracellular mechanisms, such as regulation of epigenetics. The outcomes of this study also showed that etanercept and adalimumab decreased the production of CCL2 in leukemic monocyte cell lines (THP-1 cells) and human primary monocytes (195). According to these outcomes, infliximab reduces CCL2 production *in vivo*, signifying a broader effect on inflammatory pathway activity. Moreover, regarding the limitations of anti-CCL2 monotherapies in chronic inflammatory disorders such as RA and OA, it is possible that combining anti-TNF-α and anti-CCL2 antibodies can be a potential approach in treating OA.

Another interesting therapeutic tactic in this context is using bi-specific antibodies (BsAbs), which can bind to two types of epitopes (196). The clinical assessment of bsAbs in autoimmune diseases is in progress, with both achievements (phase II trials of obexelimab in systemic lupus erythematosus) and failures (phase II trials of lutikizumab in OA and romilkimab in IPF) (197, 198). Lutikizumab (ABT-981) is a human dual variable domain Ig that can bind and inhibit IL-1α and IL-1β (199). According to the results of this trial, IL-1 inhibition was not an effective analgesic/antiinflammatory treatment in most patients with knee OA and associated synovitis, based on the limited improvement in WOMAC pain scores and lack of synovitis improvement with lutikizumab. Regarding these limitations and promising results from evaluating BsAbs in RA, designing novel BsAbs, targeting CCL2 and other inflammatory mediators such as TNF-α, IL-1β, and IL-17 may improve the effectiveness of immunotherapy in patients with OA in a synergistic fashion (200, 201). However, this approach needs more studies in the preclinical and clinical phases.

#### 5.1.4 Nanoparticle-based therapies

Targeted therapies have become increasingly common in utilizing nanoparticles as delivery vehicles (202). Nanoparticles can help therapeutic agents become more bioavailable, prolong circulation, and enhance their accumulation in the joints of arthritis patients (203). Evidence demonstrated that by employing nanomaterials to treat OA, cargo such as small-molecular drugs, nucleic acids, and peptides/proteins can be transported to inhibit its progression (204, 205). Accordingly, the therapeutic potential of CCR2 antagonists or anti-CCL2 antibodies can be enhanced by encapsulating them within nanoparticles. By minimizing systemic side effects and maximizing local concentrations of therapeutic agents within the joint, nanoparticle-based therapies offer the advantage of controlled release.

In this context, an investigation revealed that the inhibitor was investigated for treating PTOA through a unique method of releasing RS504393 slowly and continuously into injured knees using biodegradable microplates (PLs) (155). In mice with DMM/sham surgery, RS-PLs (1 mg/kg) were administered intraarticularly one week after surgery, followed by subsequent administrations at 4- and 7 weeks following surgery. Drug-free PLs (DF-PLs) or saline injections were given as controls. Results indicated that RS-PLs facilitated sustained release of CCR2 inhibitors over several weeks, with approximately 20% of the drug still available after 21 days. Both early and severe PTOA stages were significantly improved with this extended release, which reduced bone damage, synovial hyperplasia, and extracellular matrix loss (**Table 1**). However, extracellular matrix loss was less effectively mitigated. The findings highlight the importance of local sustained delivery in the optimization of CCR2-targeted therapies for PTOA.

By overcoming the challenges associated with conventional systemic drug delivery, nanoparticle-based drug delivery approaches could revolutionize the treatment landscape for OA.

## 6 Future directions and concluding notes

It is expected that targeted therapies for OA will continue to grow and innovate in the future (**Box 1**). There is potential for further advancements in research focusing on refining treatment strategies, understanding patient-specific responses, and finding novel therapeutic targets within the inflammatory cascade. Despite the promising progress in developing targeted therapies targeting the CCL2/CCR2 axis, translating preclinical successes into clinical success remains challenging. Understanding individualized treatment approaches is complex due to patient heterogeneity, disease phenotypes, and the intricate interplay among multiple signaling pathways. Moreover, in the context of chronic diseases such as arthritis, it is important to assess the long-term safety and tolerability of these interventions carefully. Future research efforts should focus on improving patient stratification strategies, understanding molecular mechanisms underlying treatment responses, and exploring combinatorial approaches to OA’s multifaceted pathology. To fully resolve the symphony of OA management, academia, industry, and regulatory agencies must collaborate to accelerate the development and approval of targeted therapies for OA.

Box 1 **Key points of clinical implications and therapeutic perspectives.**

**Figure:**
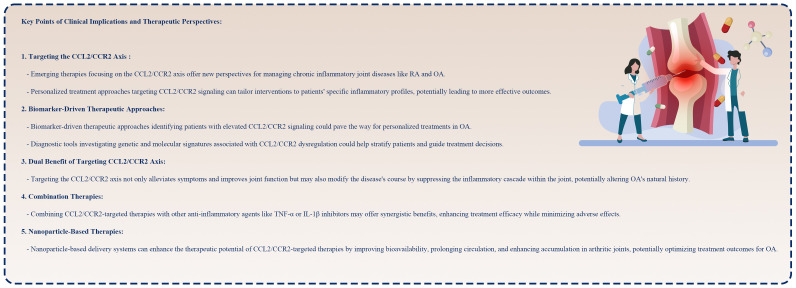

Targeting the CCL2/CCR2 axis in OA has significant clinical implications and therapeutic opportunities. In addition to personalized medicine and disease modification, these emerging strategies offer hope to millions suffering from OA through innovative drug delivery and combination therapies. As the symphony of OA management evolves, CCL2/CCR2-targeted therapies are becoming increasingly significant in alleviating the burden associated with this disease. However, the variability in outcomes underscores the complexity of OA pathology and the requirement for personalized approaches in targeting the CCL2/CCR2 axis.

## Author contributions

MA: Writing – original draft, Writing – review & editing. HK: Conceptualization, Investigation, Visualization, Writing – original draft, Writing – review & editing.

## References

[1] Houard X Goldring MB Berenbaum F. Homeostatic mechanisms in articular cartilage and role of inflammation in osteoarthritis. Curr Rheumatol Rep. (2013) 15:1–10. doi: 10.1007/s11926-013-0375-6 PMC3989071 24072604

[2] Leung GJ Rainsford K Kean WF. Osteoarthritis of the hand I: aetiology and pathogenesis, risk factors, investigation and diagnosis. J Pharm Pharmacol. (2014) 66:339–46. doi: 10.1111/jphp.12196 24329488

[3] Musumeci G Aiello FC Szychlinska MA Di Rosa M Castrogiovanni P Mobasheri A. Osteoarthritis in the XXIst century: risk factors and behaviours that influence disease onset and progression. Int J Mol Sci. (2015) 16:6093–112. doi: 10.3390/ijms16036093 PMC4394521 25785564

[4] Rainville J Bono JV Laxer EB Kim DH Lavelle JM Indahl A. Comparison of the history and physical examination for hip osteoarthritis and lumbar spinal stenosis. Spine J. (2019) 19:1009–18. doi: 10.1016/j.spinee.2019.01.006 30708114

[5] Swagerty DL Jr. Hellinger D. Radiographic assessment of osteoarthritis. Am Family Physician. (2001) 64:279–87. 11476273

[6] Kamaruzaman H Kinghorn P Oppong R. Cost-effectiveness of surgical interventions for the management of osteoarthritis: a systematic review of the literature. BMC musculoskeletal Disord. (2017) 18:1–17. doi: 10.1186/s12891-017-1540-2 PMC5424321 28486957

[7] Kamiab Z Khorramdelazad H Kafi M Jafarzadeh A Mohammadi-Shahrokhi V Bagheri-Hosseinabadi Z. Role of Interleukin-17 family cytokines in disease severity of patients with knee osteoarthritis. Adv Rheumatol. (2024) 64:1–8. doi: 10.1186/s42358-024-00351-5 38268022 PMC13488753

[8] Abassifard M Khorramdelazad H Rezaee S Jafarzadeh A. Higher circulating concentration of Interleukin-38 in patients with knee osteoarthritis: its association with disease severity. Iranian J Allergy Asthma Immunol. (2021) 20:114–9. doi: 10.18502/ijaai.v20i1.5418 33639627

[9] Lana J Rodrigues BL. Osteoarthritis as a chronic inflammatory disease: a review of the inflammatory markers. Osteoarthritis Biomarkers treatments. (2019).

[10] Raghu H Lepus CM Wang Q Wong HH Lingampalli N Oliviero F. CCL2/CCR2, but not CCL5/CCR5, mediates monocyte recruitment, inflammation and cartilage destruction in osteoarthritis. Ann rheumatic Dis. (2016) 76:914–22. 10.1136/annrheumdis-2016-210426 PMC5834918 27965260

[11] Yao Q Wu X Tao C Gong W Chen M Qu M. Osteoarthritis: pathogenic signaling pathways and therapeutic targets. Signal Transduction Targeted Ther. (2023) 8:56. doi: 10.1038/s41392-023-01330-w PMC9898571 36737426

[12] Haubruck P Pinto MM Moradi B Little CB Gentek R. Monocytes, macrophages, and their potential niches in synovial joints–therapeutic targets in post-traumatic osteoarthritis? Front Immunol. (2021) 12:763702. doi: 10.3389/fimmu.2021.763702 34804052 PMC8600114

[13] Scanzello CR. Chemokines and inflammation in osteoarthritis: insights from patients and animal models. J Orthopaedic Res. (2017) 35:735–9. doi: 10.1002/jor.23471 PMC5912941 27808445

[14] O'Brien KM Wiggers J Williams A Campbell E Wolfenden L Yoong S. Randomised controlled trial of referral to a telephone-based weight management and healthy lifestyle programme for patients with knee osteoarthritis who are overweight or obese: a study protocol. BMJ Open. (2016) 6:e010203. doi: 10.1136/bmjopen-2015-010203 PMC4785282 26940110

[15] Oo WM Yu SP-C Daniel MS Hunter DJ. Disease-modifying drugs in osteoarthritis: current understanding and future therapeutics. Expert Opin emerging Drugs. (2018) 23:331–47. doi: 10.1080/14728214.2018.1547706 30415584

[16] Latourte A Kloppenburg M Richette P. Emerging pharmaceutical therapies for osteoarthritis. Nat Rev Rheumatol. (2020) 16:673–88. doi: 10.1038/s41584-020-00518-6 33122845

[17] Buckwalter JA Mankin HJ Grodzinsky AJ. Articular cartilage and osteoarthritis. Instructional Course Lectures. (2005) 54:465. 15952258

[18] Lockwood W. Osteoarthritis (Degenerative Joint Disease). (2021). Available at: http://www.caregiversflorida.org/courses/coursematerial-247.pdf.

[19] Menz HB Zammit GV Landorf KB Munteanu SE. Plantar calcaneal spurs in older people: longitudinal traction or vertical compression? J Foot Ankle Res. (2008) 1:1–7. doi: 10.1186/1757-1146-1-7 18822162 PMC2553779

[20] Duclos M. Osteoarthritis, obesity and type 2 diabetes: the weight of waist circumference. Ann Phys Rehabil Med. (2016) 59:157–60. doi: 10.1016/j.rehab.2016.04.002 27211819

[21] Guilak F. Biomechanical factors in osteoarthritis. Best Pract Res Clin Rheumatol. (2011) 25:815–23. doi: 10.1016/j.berh.2011.11.013 PMC3266544 22265263

[22] Katz JN Arant KR Loeser RF. Diagnosis and treatment of hip and knee osteoarthritis: a review. Jama. (2021) 325:568–78. doi: 10.1001/jama.2020.22171 PMC8225295 33560326

[23] Khoja SS Almeida GJ Freburger JK. Recommendation rates for physical therapy, lifestyle counseling, and pain medications for managing knee osteoarthritis in ambulatory care settings: a cross-sectional analysis of the National Ambulatory Care Survey (2007–2015). Arthritis Care Res. (2020) 72:184–92. doi: 10.1002/acr.24064 31595710

[24] Lieberthal J Sambamurthy N Scanzello CR. Inflammation in joint injury and post-traumatic osteoarthritis. Osteoarthritis cartilage. (2015) 23:1825–34. doi: 10.1016/j.joca.2015.08.015 PMC4630675 26521728

[25] Anderson DD Chubinskaya S Guilak F Martin JA Oegema TR Olson SA. Post-traumatic osteoarthritis: improved understanding and opportunities for early intervention. J orthopaedic Res. (2011) 29:802–9. doi: 10.1002/jor.21359 PMC3082940 21520254

[26] Little CB Hunter DJ. Post-traumatic osteoarthritis: from mouse models to clinical trials. Nat Rev Rheumatol. (2013) 9:485–97. doi: 10.1038/nrrheum.2013.72 23689231

[27] Pitsillides AA Beier F. Cartilage biology in osteoarthritis—lessons from developmental biology. Nat Rev Rheumatol. (2011) 7:654–63. doi: 10.1038/nrrheum.2011.129 21947178

[28] Akkiraju H Nohe A. Role of chondrocytes in cartilage formation, progression of osteoarthritis and cartilage regeneration. J Dev Biol. (2015) 3:177–92. doi: 10.3390/jdb3040177 PMC4916494 27347486

[29] Sutton S Clutterbuck A Harris P Gent T Freeman S Foster N. The contribution of the synovium, synovial derived inflammatory cytokines and neuropeptides to the pathogenesis of osteoarthritis. veterinary J. (2009) 179:10–24. doi: 10.1016/j.tvjl.2007.08.013 17911037

[30] Han D Fang Y Tan X Jiang H Gong X Wang X. The emerging role of fibroblast-like synoviocytes-mediated synovitis in osteoarthritis: An update. J Cell Mol Med. (2020) 24:9518–32. doi: 10.1111/jcmm.15669 PMC7520283 32686306

[31] Tanaka Y Nakayamada S Okada Y. Osteoblasts and osteoclasts in bone remodeling and inflammation. Curr Drug Targets-Inflammation Allergy. (2005) 4:325–8. doi: 10.2174/1568010054022015 16101541

[32] Finzel S Sahinbegovic E Kocijan R Engelke K Englbrecht M Schett G. Inflammatory bone spur formation in psoriatic arthritis is different from bone spur formation in hand osteoarthritis. Arthritis Rheumatol. (2014) 66:2968–75. doi: 10.1002/art.38794 25048110

[33] Furusato B Rhim JS. CXCR4 and cancer. Chemokine Receptors Cancer. (2009), 31–45.

[34] Botter SM van Osch GJ Clockaerts S Waarsing JH Weinans H van Leeuwen JP. Osteoarthritis induction leads to early and temporal subchondral plate porosity in the tibial plateau of mice: an in vivo microfocal computed tomography study. Arthritis Rheumatism. (2011) 63:2690–9. doi: 10.1002/art.30307 21360519

[35] Zhen G Wen C Jia X Li Y Crane JL Mears SC. Inhibition of TGF-β signaling in mesenchymal stem cells of subchondral bone attenuates osteoarthritis. Nat Med. (2013) 19:704–12. doi: 10.1038/nm.3143 PMC3676689 23685840

[36] Klose-Jensen R Hartlev LB Boel LWT Laursen MB Stengaard-Pedersen K Keller KK. Subchondral bone turnover, but not bone volume, is increased in early stage osteoarthritic lesions in the human hip joint. Osteoarthritis cartilage. (2015) 23:2167–73. doi: 10.1016/j.joca.2015.06.001 26074361

[37] Bettica P Cline G Hart DJ Meyer J Spector TD. Evidence for increased bone resorption in patients with progressive knee osteoarthritis: longitudinal results from the Chingford study. Arthritis Rheumatism. (2002) 46:3178–84. doi: 10.1002/art.10630 12483721

[38] Jiao K Zhang M Niu L Yu S Zhen G Xian L. Overexpressed TGF-β in subchondral bone leads to mandibular condyle degradation. J Dental Res. (2014) 93:140–7. doi: 10.1177/0022034513513034 24309371

[39] Zhen G Cao X. Targeting TGFβ signaling in subchondral bone and articular cartilage homeostasis. Trends Pharmacol Sci. (2014) 35:227–36. doi: 10.1016/j.tips.2014.03.005 PMC4058854 24745631

[40] Li G Yin J Gao J Cheng TS Pavlos NJ Zhang C. Subchondral bone in osteoarthritis: insight into risk factors and microstructural changes. Arthritis Res Ther. (2013) 15:1–12. doi: 10.1186/ar4405 PMC4061721 24321104

[41] Geurts J Patel A Hirschmann MT Pagenstert GI Müller‐Gerbl M Valderrabano V. Elevated marrow inflammatory cells and osteoclasts in subchondral osteosclerosis in human knee osteoarthritis. J Orthopaedic Res. (2016) 34:262–9. doi: 10.1002/jor.23009 26250062

[42] Roberts CA Dickinson AK Taams LS. The interplay between monocytes/macrophages and CD4+ T cell subsets in rheumatoid arthritis. Front Immunol. (2015) 6:571. doi: 10.3389/fimmu.2015.00571 26635790 PMC4652039

[43] Wu C-L Harasymowicz NS Klimak MA Collins KH Guilak F. The role of macrophages in osteoarthritis and cartilage repair. Osteoarthritis cartilage. (2020) 28:544–54. doi: 10.1016/j.joca.2019.12.007 PMC7214213 31926267

[44] Schulze-Tanzil G. Intraarticular ligament degeneration is interrelated with cartilage and bone destruction in osteoarthritis. Cells. (2019) 8:990. doi: 10.3390/cells8090990 31462003 PMC6769780

[45] Marsh LJ Kemble S Nisa Reis P Singh R Croft AP. Fibroblast pathology in inflammatory joint disease. Immunol Rev. (2021) 302:163–83. doi: 10.1111/imr.12986 34096076

[46] Masoumi M Bashiri H Khorramdelazad H Barzaman K Hashemi N Sereshki HA. Destructive roles of fibroblast-like synoviocytes in chronic inflammation and joint damage in rheumatoid arthritis. Inflammation. (2021) 44:466–79. doi: 10.1007/s10753-020-01371-1 33113036

[47] Geyer M Schönfeld C. Novel insights into the pathogenesis of osteoarthritis. Curr Rheumatol Rev. (2018) 14:98–107. doi: 10.2174/1573397113666170807122312 28782470

[48] Woodell-May JE Sommerfeld SD. Role of inflammation and the immune system in the progression of osteoarthritis. J Orthopaedic Research®. (2020) 38:253–7. doi: 10.1002/jor.24457 31469192

[49] Berenbaum F. Osteoarthritis as an inflammatory disease (osteoarthritis is not osteoarthrosis!). Osteoarthritis cartilage. (2013) 21:16–21. doi: 10.1016/j.joca.2012.11.012 23194896

[50] Navegantes KC de Souza Gomes R Pereira PAT Czaikoski PG Azevedo CHM Monteiro MC. Immune modulation of some autoimmune diseases: the critical role of macrophages and neutrophils in the innate and adaptive immunity. J Trans Med. (2017) 15:1–21. doi: 10.1186/s12967-017-1141-8 PMC5312441 28202039

[51] Barreto G Manninen M Eklund KK. Osteoarthritis and toll-like receptors: when innate immunity meets chondrocyte apoptosis. Biology. (2020) 9:65. doi: 10.3390/biology9040065 32235418 PMC7235883

[52] Miller RJ Malfait A-M Miller RE. The innate immune response as a mediator of osteoarthritis pain. Osteoarthritis Cartilage. (2020) 28:562–71. doi: 10.1016/j.joca.2019.11.006 PMC6951330 31862470

[53] Newton K Dixit VM. Signaling in innate immunity and inflammation. Cold Spring Harbor Perspect Biol. (2012) 4:a006049. doi: 10.1101/cshperspect.a006049 PMC3282411 22296764

[54] Kim HA Cho ML Choi HY Yoon CS Jhun JY Oh HJ. The catabolic pathway mediated by Toll-like receptors in human osteoarthritic chondrocytes. Arthritis Rheumatism. (2006) 54:2152–63. doi: 10.1002/art.21951 16802353

[55] Gobezie R Kho A Krastins B Sarracino D Thornhill T Chase M. High abundance synovial fluid proteome: distinct profiles in health and osteoarthritis. Arthritis Res Ther. (2007) 9:R36. doi: 10.1186/ar2172 17407561 PMC1906814

[56] Zhu X Zhu J. CD4 T helper cell subsets and related human immunological disorders. Int J Mol Sci. (2020) 21:8011. doi: 10.3390/ijms21218011 33126494 PMC7663252

[57] Cantor H Boyse E. Regulation of cellular and humoral immune responses by T-cell subclasses. In: Cold Spring Harbor symposia on quantitative biology. Cold Spring Harbor Laboratory Press (1977). doi: 10.1101/SQB.1977.041.01.006 302190

[58] Shapouri-Moghaddam A Mohammadian S Vazini H Taghadosi M Esmaeili SA Mardani F. Macrophage plasticity, polarization, and function in health and disease. J Cell Physiol. (2018) 233:6425–40. doi: 10.1002/jcp.26429 PMC13482560 29319160

[59] Murray PJ. Macrophage polarization. Annu Rev Physiol. (2017) 79:541–66. doi: 10.1146/annurev-physiol-022516-034339 27813830

[60] Qi C Shan Y Wang J Ding F Zhao D Yang T. Circulating T helper 9 cells and increased serum interleukin-9 levels in patients with knee osteoarthritis. Clin Exp Pharmacol Physiol. (2016) 43:528–34. doi: 10.1111/1440-1681.12567 26926842

[61] Li Y-s Luo W Zhu S-A Lei G-H. T cells in osteoarthritis: alterations and beyond. Front Immunol. (2017) 8:356. doi: 10.3389/fimmu.2017.00356 28424692 PMC5371609

[62] Robinson WH Lepus CM Wang Q Raghu H Mao R Lindstrom TM. Low-grade inflammation as a key mediator of the pathogenesis of osteoarthritis. Nat Rev Rheumatol. (2016) 12:580–92. doi: 10.1038/nrrheum.2016.136 PMC5500215 27539668

[63] Scanzello CR Goldring SR. The role of synovitis in osteoarthritis pathogenesis. Bone. (2012) 51:249–57. doi: 10.1016/j.bone.2012.02.012 PMC3372675 22387238

[64] Huang Z Chen J Li B Zeng B Chou C-H Zheng X. Faecal microbiota transplantation from metabolically compromised human donors accelerates osteoarthritis in mice. Ann rheumatic Dis. (2020) 79:646–56. doi: 10.1136/annrheumdis-2019-216471 PMC7384301 32205337

[65] Tsai JC Casteneda G Lee A Dereschuk K Li WT Chakladar J. Identification and characterization of the intra-articular microbiome in the osteoarthritic knee. Int J Mol Sci. (2020) 21:8618. doi: 10.3390/ijms21228618 33207573 PMC7697780

[66] Raghu H Lepus CM Wang Q Wong HH Lingampalli N Oliviero F. CCL2/CCR2, but not CCL5/CCR5, mediates monocyte recruitment, inflammation and cartilage destruction in osteoarthritis. Ann rheumatic Dis. (2017) 76:914–22. doi: 10.1136/annrheumdis-2016-210426 PMC5834918 27965260

[67] Zarebska JM Chanalaris A Driscoll C Burleigh A Miller R Malfait A. CCL2 and CCR2 regulate pain-related behaviour and early gene expression in post-traumatic murine osteoarthritis but contribute little to chondropathy. Osteoarthritis cartilage. (2017) 25:406–12. doi: 10.1016/j.joca.2016.10.008 PMC5358501 27746376

[68] Agarwal S Misra R Aggarwal A. Induction of metalloproteinases expression by TLR ligands in human fibroblast like synoviocytes from juvenile idiopathic arthritis patients. Indian J Med Res. (2010) 131:771–9. 20571165

[69] Nie F Ding F Chen B Huang S Liu Q Xu C. Dendritic cells aggregate inflammation in experimental osteoarthritis through a toll-like receptor (TLR)-dependent machinery response to challenges. Life Sci. (2019) 238:116920. doi: 10.1016/j.lfs.2019.116920 31610189

[70] Huang Z Kraus VB. Does lipopolysaccharide-mediated inflammation have a role in OA? Nat Rev Rheumatol. (2016) 12:123–9. doi: 10.1038/nrrheum.2015.158 PMC4930555 26656661

[71] Dunn CM Velasco C Rivas A Andrews M Garman C Jacob PB. Identification of cartilage microbial DNA signatures and associations with knee and hip osteoarthritis. Arthritis Rheumatol. (2020) 72:1111–22. doi: 10.1002/art.41210 PMC7336391 31961065

[72] Zhao Y Chen B Li S Yang L Zhu D Wang Y. Detection and characterization of bacterial nucleic acids in culture-negative synovial tissue and fluid samples from rheumatoid arthritis or osteoarthritis patients. Sci Rep. (2018) 8:14305. doi: 10.1038/s41598-018-32675-w 30250232 PMC6155189

[73] RF L. Association of increased serum lipopolysaccharide, but not microbial dysbiosis, with obesity‐related osteoarthritis. Arthritis Rheumatol. (2022) 74:227–36. doi: 10.1002/art.41955 PMC8795472 34423918

[74] Thote S Gorella P Arya S Mourya A Devangan P Jyothi VGS. Synergy between cyclooxygenase-2 inhibitors and hyaluronic acid in the treatment of osteoarthritis: Illumination of signaling cascade, nanotechnology-driven delivery strategies and future prospects. J Drug Deliv Sci Technol. (2024) 92:105380. doi: 10.1016/j.jddst.2024.105380

[75] Kennedy O Kitson A Okpara C Chow LW Gonzalez-Fernandez T. Immunomodulatory strategies for cartilage regeneration in osteoarthritis. Tissue Eng Part A. (2024) 30:259–271. doi: 10.1089/ten.tea.2023.0255 38126327

[76] DuoYi Li M Rong Hang M Lang Meng M Zhigang Zhao M Chunmei Zhao M. Co-treatment with oral duloxetine and intraarticular injection of corticosteroid plus hyaluronic acid reduces pain in the treatment of knee osteoarthritis. Pain Physician. (2024) 27:E45–53. 38285030

[77] Xiao P Han X Huang Y Yang J Chen L Cai Z. Reprogramming macrophages via immune cell mobilized hydrogel microspheres for osteoarthritis treatments. Bioactive Materials. (2024) 32:242–59. doi: 10.1016/j.bioactmat.2023.09.010 PMC10589729 37869722

[78] Wen X Fang G Li H Jiang Z Du X Liao Z. CircIRAK3 exerts negative feedback regulation on inflammation by binding to HNRNP U and destabilizing proinflammatory cytokine mRNA in osteoarthritis and chondrogenesis. Int J Biol Macromolecules. (2024) 256:128453. doi: 10.1016/j.ijbiomac.2023.128453 38016613

[79] Tan Z Chen R Lin H Wang H Liu H. The identification of immune-related biomarkers for osteoarthritis immunotherapy based on single-cell RNA sequencing analysis. Genet Research 2023. (2023) 2023:e17. doi: 10.1155/2023/5574636 PMC10030227 36960385

[80] Chevalier X Ravaud P Maheu E Baron G Rialland A Vergnaud P. Adalimumab in patients with hand osteoarthritis refractory to analgesics and NSAIDs: a randomised, multicentre, double-blind, placebo-controlled trial. Ann rheumatic Dis. (2015) 74:1697–705. doi: 10.1136/annrheumdis-2014-205348 24817417

[81] Richette P Latourte A Sellam J Wendling D Piperno M Goupille P. Efficacy of tocilizumab in patients with hand osteoarthritis: double blind, randomised, placebo-controlled, multicentre trial. Ann rheumatic Dis. (2021) 80:349–55. doi: 10.1136/annrheumdis-2020-218547 33055078

[82] Bannwarth B. Acetaminophen or NSAIDs for the treatment of osteoarthritis. Best Pract Res Clin Rheumatol. (2006) 20:117–29. doi: 10.1016/j.berh.2005.09.004 16483911

[83] Yucesoy B Charles LE Baker B Burchfiel CM. Occupational and genetic risk factors for osteoarthritis: a review. Work. (2015) 50:261–73. doi: 10.3233/WOR-131739 PMC4562436 24004806

[84] O’Connor T Borsig L Heikenwalder M. CCL2-CCR2 signaling in disease pathogenesis. Endocrine Metab Immune Disorders-Drug Targets. (2015) 15:105–18. doi: 10.2174/1871530315666150316120920 25772168

[85] Gholamalizadeh H Ensan B Sukhorukov VN Sahebkar A. Targeting the CCL2-CCR2 signaling pathway: potential implications of statins beyond cardiovascular diseases. J Pharm Pharmacol. (2023) 76:138–153. doi: 10.1093/jpp/rgad112 38127312

[86] Zhu S Liu M Bennett S Wang Z Pfleger KDG Xu J. The molecular structure and role of CCL2 (MCP-1) and C-C chemokine receptor CCR2 in skeletal biology and diseases. J Cell Physiol. (2021) 236:7211–22. doi: 10.1002/jcp.30375 33782965

[87] Oberthür D Achenbach J Gabdulkhakov A Buchner K Maasch C Falke S. Crystal structure of a mirror-image L-RNA aptamer (Spiegelmer) in complex with the natural L-protein target CCL2. Nat Commun. (2015) 6:6923. doi: 10.1038/ncomms7923 25901662 PMC4423205

[88] Yuan J. CCR2: A characteristic chemokine receptor in normal and pathological intestine. Cytokine. (2023) 169:156292. doi: 10.1016/j.cyto.2023.156292 37437448

[89] Huang B Lei Z Zhao J Gong W Liu J Chen Z. CCL2/CCR2 pathway mediates recruitment of myeloid suppressor cells to cancers. Cancer Lett. (2007) 252:86–92. doi: 10.1016/j.canlet.2006.12.012 17257744

[90] Moadab F Khorramdelazad H Abbasifard M. Role of CCL2/CCR2 axis in the immunopathogenesis of rheumatoid arthritis: Latest evidence and therapeutic approaches. Life Sci. (2021) 269:119034. doi: 10.1016/j.lfs.2021.119034 33453247

[91] Ranjbar M Rahimi A Baghernejadan Z Ghorbani A Khorramdelazad H. Role of CCL2/CCR2 axis in the pathogenesis of COVID-19 and possible Treatments: All options on the Table. Int Immunopharmacol. (2022) 113:109325. doi: 10.1016/j.intimp.2022.109325 36252475 PMC9561120

[92] Vakilian A Khorramdelazad H Heidari P Rezaei ZS Hassanshahi G. CCL2/CCR2 signaling pathway in glioblastoma multiforme. Neurochemistry Int. (2017) 103:1–7. doi: 10.1016/j.neuint.2016.12.013 28025034

[93] Behfar S Hassanshahi G Nazari A Khorramdelazad H. A brief look at the role of monocyte chemoattractant protein-1 (CCL2) in the pathophysiology of psoriasis. Cytokine. (2018) 110:226–31. doi: 10.1016/j.cyto.2017.12.010 29277337

[94] Taghavi Y Hassanshahi G Kounis NG Koniari I Khorramdelazad H. Monocyte chemoattractant protein-1 (MCP-1/CCL2) in diabetic retinopathy: latest evidence and clinical considerations. J Cell communication Signaling. (2019) 13:451–62. doi: 10.1007/s12079-018-00500-8 PMC6946768 30607767

[95] Lu Y Jiang B-C Cao D-L Zhang Z-J Zhang X Ji R-R. TRAF6 upregulation in spinal astrocytes maintains neuropathic pain by integrating TNF-α and IL-1β signaling. PAIN®. (2014) 155:2618–29. doi: 10.1016/j.pain.2014.09.027 PMC4250420 25267210

[96] Puntambekar SS Davis DS Hawel L III Crane J Byus CV Carson MJ. LPS-induced CCL2 expression and macrophage influx into the murine central nervous system is polyamine-dependent. Brain behavior Immun. (2011) 25:629–39. doi: 10.1016/j.bbi.2010.12.016 PMC3081407 21237263

[97] Thompson WL Van Eldik LJ. Inflammatory cytokines stimulate the chemokines CCL2/MCP-1 and CCL7/MCP-7 through NFκB and MAPK dependent pathways in rat astrocytes. Brain Res. (2009) 1287:47–57. doi: 10.1016/j.brainres.2009.06.081 19577550 PMC2725204

[98] Chen C-Y Fuh L-J Huang C-C Hsu C-J Su C-M Liu S-C. Enhancement of CCL2 expression and monocyte migration by CCN1 in osteoblasts through inhibiting miR-518a-5p: implication of rheumatoid arthritis therapy. Sci Rep. (2017) 7:421. doi: 10.1038/s41598-017-00513-0 28341837 PMC5428676

[99] Emre Y Imhof BA. Matricellular protein CCN1/CYR61: a new player in inflammation and leukocyte trafficking. Seminars in immunopathology. Springer (2014), 253–9. doi: 10.1007/s00281-014-0420-1 24638890

[100] MacDonald IJ Huang C-C Liu S-C Lin Y-Y Tang C-H. Targeting CCN proteins in rheumatoid arthritis and osteoarthritis. Int J Mol Sci. (2021) 22:4340. doi: 10.3390/ijms22094340 33919365 PMC8122640

[101] Panganiban RP Vonakis BM Ishmael FT Stellato C. Coordinated post-transcriptional regulation of the chemokine system: messages from CCL2. J Interferon Cytokine Res. (2014) 34:255–66. doi: 10.1089/jir.2013.0149 PMC3976576 24697203

[102] Chen Y Liu S Wu L Liu Y Du J Luo Z. Epigenetic regulation of chemokine (CC‐motif) ligand 2 in inflammatory diseases. Cell Proliferation. (2023) 56:e13428. doi: 10.1111/cpr.13428 36872292 PMC10334270

[103] Feria M Díaz-González F. The CCR2 receptor as a therapeutic target. Expert Opin Ther Patents. (2006) 16:49–57. doi: 10.1517/13543776.16.1.49

[104] Sobhia ME Singh R Kare P Chavan S. Rational design of CCR2 antagonists: a survey of computational studies. Expert Opin Drug Discovery. (2010) 5:543–57. doi: 10.1517/17460441.2010.482559 22823166

[105] Gschwandtner M Derler R Midwood KS. More than just attractive: how CCL2 influences myeloid cell behavior beyond chemotaxis. Front Immunol. (2019) 10:2759. doi: 10.3389/fimmu.2019.02759 31921102 PMC6923224

[106] Sierra-Filardi E Nieto C Dominguez-Soto A Barroso R Sánchez-Mateos P Puig-Kroger A. CCL2 shapes macrophage polarization by GM-CSF and M-CSF: identification of CCL2/CCR2-dependent gene expression profile. J Immunol. (2014) 192:3858–67. doi: 10.4049/jimmunol.1302821 24639350

[107] Jimenez F Quinones MP Martinez HG Estrada CA Clark K Garavito E. CCR2 plays a critical role in dendritic cell maturation: possible role of CCL2 and NF-κB. J Immunol. (2010) 184:5571–81. doi: 10.4049/jimmunol.0803494 PMC2929965 20404272

[108] Bakos E Thaiss CA Kramer MP Cohen S Radomir L Orr I. CCR2 regulates the immune response by modulating the interconversion and function of effector and regulatory T cells. J Immunol. (2017) 198:4659–71. doi: 10.4049/jimmunol.1601458 28507030

[109] Yeung YT Aziz F Guerrero-Castilla A Arguelles S. Signaling pathways in inflammation and anti-inflammatory therapies. Curr Pharm design. (2018) 24:1449–84. doi: 10.2174/1381612824666180327165604 29589535

[110] Papathanasiou I Malizos KN Tsezou A. Bone morphogenetic protein-2-induced Wnt/β-catenin signaling pathway activation through enhanced low-density-lipoprotein receptor-related protein 5 catabolic activity contributes to hypertrophy in osteoarthritic chondrocytes. Arthritis Res Ther. (2012) 14:1–14. doi: 10.1186/ar3805 22513174 PMC3446456

[111] Wu L Huang X Li L Huang H Xu R Luyten W. Insights on biology and pathology of HIF-1α/-2α, TGFα/BMP, Wnt/β-catenin, and NF-κB pathways in osteoarthritis. Curr Pharm design. (2012) 18:3293–312. doi: 10.2174/1381612811209023293 22646092

[112] Cheleschi S De Palma A Pecorelli A Pascarelli NA Valacchi G Belmonte G. Hydrostatic pressure regulates microRNA expression levels in osteoarthritic chondrocyte cultures via the Wnt/β-catenin pathway. Int J Mol Sci. (2017) 18:133. doi: 10.3390/ijms18010133 28085114 PMC5297766

[113] Meo Burt P. High Molecular Weight Isoforms of FGF2 Contribute to Osteoarthritis by FGF23 mediated Wnt/β-catenin Signaling. (dissertations) (2018).

[114] Longobardi L Jordan JM Shi XA Renner JB Schwartz TA Nelson AE. Associations between the chemokine biomarker CCL2 and knee osteoarthritis outcomes: the Johnston County Osteoarthritis Project. Osteoarthritis cartilage. (2018) 26:1257–61. doi: 10.1016/j.joca.2018.04.012 PMC6098742 29723633

[115] Lin Y-M Hsu C-J Liao Y-Y Chou M-C Tang C-H. The CCL2/CCR2 axis enhances vascular cell adhesion molecule-1 expression in human synovial fibroblasts. PloS One. (2012) 7:e49999. doi: 10.1371/journal.pone.0049999 23185512 PMC3503714

[116] Zhang Y Liu D Vithran DTA Kwabena BR Xiao W Li Y. CC chemokines and receptors in osteoarthritis: new insights and potential targets. Arthritis Res Ther. (2023) 25:113. doi: 10.1186/s13075-023-03096-6 37400871 PMC10316577

[117] Luo H Li L Han S Liu T. The role of monocyte/macrophage chemokines in pathogenesis of osteoarthritis: A review. Int J Immunogenetics. (2024) 51:130–142. doi: 10.1111/iji.12664 38462560

[118] Paish HL Kalson NS Smith GR del Carpio Pons A Baldock TE Smith N. Fibroblasts promote inflammation and pain via IL-1α induction of the monocyte chemoattractant chemokine (CC motif) ligand 2. Am J Pathol. (2018) 188:696–714. doi: 10.1016/j.ajpath.2017.11.007 29248462 PMC5842035

[119] Griffin TM Scanzello CR. Innate inflammation and synovial macrophages in osteoarthritis pathophysiology. Clin Exp Rheumatol. (2019) 37:57–63. PMC6842324 31621560

[120] Bondeson J Blom AB Wainwright S Hughes C Caterson B Van Den Berg WB. The role of synovial macrophages and macrophage-produced mediators in driving inflammatory and destructive responses in osteoarthritis. INTECH Open Access Publisher (2012). 10.1002/art.27290 20187160

[121] van den Bosch MH Blom AB Schelbergen RF Koenders MI van de Loo FA van den Berg WB. Alarmin S100A9 induces proinflammatory and catabolic effects predominantly in the M1 macrophages of human osteoarthritic synovium. J Rheumatol. (2016) 43:1874–84. doi: 10.3899/jrheum.160270 27481901

[122] Schelbergen R Geven E Van Den Bosch M Eriksson H Leanderson T Vogl T. Prophylactic treatment with S100A9 inhibitor paquinimod reduces pathology in experimental collagenase-induced osteoarthritis. Ann rheumatic Dis. (2015) 74:2254–8. doi: 10.1136/annrheumdis-2014-206517 25969431

[123] Minguzzi M Cetrullo S D’Adamo S Silvestri Y Flamigni F Borzì RM. Emerging players at the intersection of chondrocyte loss of maturational arrest, oxidative stress, senescence and low-grade inflammation in osteoarthritis. Oxid Med Cell Longevity. (2018) 51:130–42. doi: 10.1155/2018/3075293 PMC5828476 29599894

[124] Longobardi L Temple JD Tagliafierro L Willcockson H Esposito A D'Onofrio N. Role of the CC chemokine receptor-2 in a murine model of injury-induced osteoarthritis. Osteoarthritis cartilage. (2017) 25:914–25. doi: 10.1016/j.joca.2016.11.004 PMC5430000 27856294

[125] Willcockson H Ozkan H Chubinskaya S Loeser RF Longobardi L. CCL2 induces articular chondrocyte MMP expression through ERK and p38 signaling pathways. Osteoarthritis Cartilage Open. (2021) 3:100136. doi: 10.1016/j.ocarto.2020.100136 36475068 PMC9718225

[126] Willcockson H Ozkan H Valdés-Fernández J Arbeeva L Mucahit E Musawwir L. CC-chemokine receptor-2 expression in osteoblasts contributes to cartilage and bone damage during post-traumatic osteoarthritis. Biomolecules. (2023) 13:891. doi: 10.3390/biom13060891 37371471 PMC10296290

[127] Yuan GH Masuko‐Hongo K Sakata M Tsuruha JI Onuma H Nakamura H. The role of C‐C chemokines and their receptors in osteoarthritis. Arthritis Rheumatism. (2001) 44:1056–70. doi: 10.1002/(ISSN)1529-0131 11352237

[128] Li L Jiang B-E. Serum and synovial fluid chemokine ligand 2/monocyte chemoattractant protein 1 concentrations correlates with symptomatic severity in patients with knee osteoarthritis. Ann Clin Biochem. (2015) 52:276–82. doi: 10.1177/0004563214545117 25005456

[129] Haraden CA Huebner JL Hsueh M-F Li Y-J Kraus VB. Synovial fluid biomarkers associated with osteoarthritis severity reflect macrophage and neutrophil related inflammation. Arthritis Res Ther. (2019) 21:1–9. doi: 10.1186/s13075-019-1923-x 31196179 PMC6567574

[130] Boniakowski AE Kimball AS Joshi A Schaller M Davis FM denDekker A. Murine macrophage chemokine receptor CCR2 plays a crucial role in macrophage recruitment and regulated inflammation in wound healing. Eur J Immunol. (2018) 48:1445–55. doi: 10.1002/eji.201747400 PMC6371802 29879295

[131] Chow YY Chin K-Y. The role of inflammation in the pathogenesis of osteoarthritis. Mediators Inflammation. (2020) 2020. doi: 10.1155/2020/8293921 PMC7072120 32189997

[132] Zhu J Jia X Xiao G Kang Y Partridge NC Qin L. EGF-like ligands stimulate osteoclastogenesis by regulating expression of osteoclast regulatory factors by osteoblasts: implications for osteolytic bone metastases. J Biol Chem. (2007) 282:26656–65. doi: 10.1074/jbc.M705064200 17636266

[133] Harris Q Seto J O'Brien K Lee PS Kondo C Heard BJ. Monocyte chemotactic protein-1 inhibits chondrogenesis of synovial mesenchymal progenitor cells: an in vitro study. Stem Cells. (2013) 31:2253–65. doi: 10.1002/stem.1477 23836536

[134] Appleton CTG McErlain DD Pitelka V Schwartz N Bernier SM Henry JL. Forced mobilization accelerates pathogenesis: characterization of a preclinical surgical model of osteoarthritis. Arthritis Res Ther. (2007) 9:1–15. doi: 10.1186/ar2120 PMC1860072 17284317

[135] Appleton C Pitelka V Henry J Beier F. Global analyses of gene expression in early experimental osteoarthritis. Arthritis Rheumatism. (2007) 56:1854–68. doi: 10.1002/art.22711 17530714

[136] Appleton CTG Usmani SE Pest MA Pitelka V Mort JS Beier F. Reduction in disease progression by inhibition of transforming growth factor α–CCL2 signaling in experimental posttraumatic osteoarthritis. Arthritis Rheumatol. (2015) 67:2691–701. doi: 10.1002/art.39255 26138996

[137] Blanco FJ Möller I Romera M Rozadilla A Sánchez-Lázaro JA Rodríguez A. Improved prediction of knee osteoarthritis progression by genetic polymorphisms: the Arthrotest Study. Rheumatology. (2015) 54:1236–43. doi: 10.1093/rheumatology/keu478 25573839

[138] Xu Z Li J Yang H Jiang L Zhou X Huang Y. Association of CCL2 gene variants with osteoarthritis. Arch Med Res. (2019) 50:86–90. doi: 10.1016/j.arcmed.2019.05.014 31495394

[139] Hulin‐Curtis S Bidwell J Perry M. Association between CCL2 haplotypes and knee osteoarthritis. Int J immunogenetics. (2013) 40:280–3. doi: 10.1111/iji.12015 23211090

[140] Karsdal M Christiansen C Ladel C Henriksen K Kraus V Bay-Jensen A. Osteoarthritis–a case for personalized health care? Osteoarthritis Cartilage. (2014) 22:7–16. doi: 10.1016/j.joca.2013.10.018 24216058

[141] W Zimmermann H Sterzer V Sahin H. CCR1 and CCR2 antagonists. Curr topics medicinal Chem. (2014) 14:1539–52. doi: 10.2174/1568026614666140827144115 25159163

[142] Veillette CJ Jurisica I. Precision medicine for osteoarthritis. In: Osteoarthritis: Pathogenesis, Diagnosis, Available Treatments, Drug Safety, Regenerative and Precision Medicine (2015). p. 257–70. doi: 10.1007/978-3-319-19560-5_13

[143] Jüni P Reichenbach S Dieppe P. Osteoarthritis: rational approach to treating the individual. Best Pract Res Clin Rheumatol. (2006) 20:721–40. doi: 10.1016/j.berh.2006.05.002 16979535

[144] Lluch Girbés E Nijs J Torres-Cueco R Cubas López C. Pain treatment for patients with osteoarthritis and central sensitization. Phys Ther. (2013) 93:842–51. doi: 10.2522/ptj.20120253 23392185

[145] Miller RE Malfait A-M. Can we target CCR2 to treat osteoarthritis? The trick is in the timing! Osteoarthritis cartilage. (2017) 25:799–801. doi: 10.1016/j.joca.2017.01.019 28189827 PMC6006389

[146] Liu S Deng Z Chen K Jian S Zhou F Yang Y. Cartilage tissue engineering: From proinflammatory and anti−inflammatory cytokines to osteoarthritis treatments. Mol Med Rep. (2022) 25:1–15. doi: 10.3892/mmr 35088882 PMC8809050

[147] Tao Z Zhou Y Zeng B Yang X Su M. MicroRNA-183 attenuates osteoarthritic pain by inhibiting the TGFα-mediated CCL2/CCR2 signalling axis. Bone Joint Res. (2021) 10:548–57. doi: 10.1302/2046-3758.108.BJR-2019-0308.R2 PMC8414439 34463129

[148] van den Bosch MH. Osteoarthritis year in review 2020: biology. Osteoarthritis Cartilage. (2021) 29:143–50. doi: 10.1016/j.joca.2020.10.006 33242602

[149] Magni A Agostoni P Bonezzi C Massazza G Menè P Savarino V. Management of osteoarthritis: expert opinion on NSAIDs. Pain Ther. (2021) 10:783–808. doi: 10.1007/s40122-021-00260-1 33876393 PMC8586433

[150] Mathieu S Tournadre A Soubrier M Sellam J. Effect of disease-modifying anti-rheumatic drugs in osteoarthritis: A meta-analysis. Joint Bone Spine. (2022) 89:105444. doi: 10.1016/j.jbspin.2022.105444 35908643

[151] Flegar D Filipović M Šućur A Markotić A Lukač N Šisl D. Preventive CCL2/CCR2 axis blockade suppresses osteoclast activity in a mouse model of rheumatoid arthritis by reducing homing of CCR2hi osteoclast progenitors to the affected bone. Front Immunol. (2021) 12:767231. doi: 10.3389/fimmu.2021.767231 34925336 PMC8677701

[152] Na HS Lee S-Y Lee DH Woo JS Choi S-Y Cho K-H. Soluble CCR2 gene therapy controls joint inflammation, cartilage damage, and the progression of osteoarthritis by targeting MCP-1 in a monosodium iodoacetate (MIA)-induced OA rat model. J Trans Med. (2022) 20:428. doi: 10.1186/s12967-022-03515-3 PMC9503236 36138477

[153] Willcockson H Ozkan H Arbeeva L Mucahit E Musawwir L Longobardi L. Early ablation of Ccr2 in aggrecan-expressing cells following knee injury ameliorates joint damage and pain during post-traumatic osteoarthritis. Osteoarthritis Cartilage. (2022) 30:1616–30. doi: 10.1016/j.joca.2022.08.015 PMC9671864 36075514

[154] Feng M Peng H Yao R Zhang Z Mao G Yu H. Inhibition of cellular communication network factor 1 (CCN1)-driven senescence slows down cartilage inflammaging and osteoarthritis. Bone. (2020) 139:115522. doi: 10.1016/j.bone.2020.115522 32622876

[155] Ozkan H Di Francesco M Willcockson H Valdés-Fernández J Di Francesco V Granero-Moltó F. Sustained inhibition of CC-chemokine receptor-2 via intraarticular deposition of polymeric microplates in post-traumatic osteoarthritis. Drug Delivery Trans Res. (2023) 13:689–701. doi: 10.1007/s13346-022-01235-1 PMC9794532 36109442

[156] Xue C-B Wang A Han Q Zhang Y Cao G Feng H. Discovery of INCB8761/PF-4136309, a potent, selective, and orally bioavailable CCR2 antagonist. ACS medicinal Chem Lett. (2011) 2:913–8. doi: 10.1021/ml200199c PMC4018168 24900280

[157] Noel M O’Reilly EM Wolpin BM Ryan DP Bullock AJ Britten CD. Phase 1b study of a small molecule antagonist of human chemokine (CC motif) receptor 2 (PF-04136309) in combination with nab-paclitaxel/gemcitabine in first-line treatment of metastatic pancreatic ductal adenocarcinoma. Investigational New Drugs. (2020) 38:800–11. doi: 10.1007/s10637-019-00830-3 PMC7211198 31297636

[158] Loukov D Karampatos S Maly M Bowdish D. Monocyte activation is elevated in women with knee-osteoarthritis and associated with inflammation, BMI and pain. Osteoarthritis Cartilage. (2018) 26:255–63. doi: 10.1016/j.joca.2017.10.018 29128509

[159] Fumet J-D Limagne E Thibaudin M Ghiringhelli F. Immunogenic cell death and elimination of immunosuppressive cells: a double-edged sword of chemotherapy. Cancers. (2020) 12:2637. doi: 10.3390/cancers12092637 32947882 PMC7565832

[160] Li H Wu M Zhao X. Role of chemokine systems in cancer and inflammatory diseases. MedComm. (2022) 3:e147. doi: 10.1002/mco2.147 35702353 PMC9175564

[161] Duliege A-M Sleijfer S Bischof A Tan J Zhang P Seitz L. Abstract CT223: CCX872: Pharmacodynamic study of a potent and selective CCR2 antagonist in human volunteers and plans for phase Ib trial in patients with pancreatic cancer. Cancer Res. (2015) 75:CT223–3. doi: 10.1158/1538-7445.AM2015-CT223

[162] Colloca L Ludman T Bouhassira D Baron R Dickenson AH Yarnitsky D. Neuropathic pain. Nat Rev Dis Primers. (2017) 3:1–19. doi: 10.1038/nrdp.2017.2 PMC5371025 28205574

[163] Baron R. Peripheral neuropathic pain: from mechanisms to symptoms. Clin J Pain. (2000) 16:S12–20. doi: 10.1097/00002508-200006001-00004 10870735

[164] Perrot S. Osteoarthritis pain. Best Pract Res Clin Rheumatol. (2015) 29:90–7. doi: 10.1016/j.berh.2015.04.017 26267003

[165] Yu D Xu J Liu F Wang X Mao Y Zhu Z. Subchondral bone changes and the impacts on joint pain and articular cartilage degeneration in osteoarthritis. Clin Exp Rheumatol. (2016) 34:929–34. 27606839

[166] Wood MJ Miller RE Malfait A-M. The genesis of pain in osteoarthritis: inflammation as a mediator of osteoarthritis pain. Clinics geriatric Med. (2022) 38:221–38. doi: 10.1016/j.cger.2021.11.013 PMC9053380 35410677

[167] Quinones MP Estrada CA Kalkonde Y Ahuja SK Kuziel WA Mack M. The complex role of the chemokine receptor CCR2 in collagen-induced arthritis: implications for therapeutic targeting of CCR2 in rheumatoid arthritis. J Mol Med (Berl). (2005) 83:672–81. doi: 10.1007/s00109-005-0637-5 15827759

[168] Ling W Can Y Ying Meng L Man X Hu H. Berberine reduce inflammation in RA rats through MCP1/CCR2 pathway. bioRxiv. (2023), 2023.08. 09.552722.

[169] Oh SB Tran PB Gillard SE Hurley RW Hammond DL Miller RJ. Chemokines and glycoprotein120 produce pain hypersensitivity by directly exciting primary nociceptive neurons. J Neurosci. (2001) 21:5027–35. doi: 10.1523/JNEUROSCI.21-14-05027.2001 PMC6762869 11438578

[170] Taskinen HS Röyttä M. Increased expression of chemokines (MCP‐1, MIP‐1α, RANTES) after peripheral nerve transection. J Peripheral Nervous System. (2000) 5:75–81. doi: 10.1046/j.1529-8027.2000.00009.x 10905466

[171] Bhangoo S Ren D Miller RJ Henry KJ Lineswala J Hamdouchi C. Delayed functional expression of neuronal chemokine receptors following focal nerve demyelination in the rat: a mechanism for the development of chronic sensitization of peripheral nociceptors. Mol Pain. (2007) 3:1744–8069-3-38. doi: 10.1186/1744-8069-3-38 PMC2228278 18076762

[172] Cherney RJ Anjanappa P Selvakumar K Batt DG Brown GD Rose AV. BMS-813160: a potent CCR2 and CCR5 dual antagonist selected as a clinical candidate. ACS Medicinal Chem Lett. (2021) 12:1753–8. doi: 10.1021/acsmedchemlett.1c00373 PMC8591721 34795864

[173] Zhao Q Murtaza A Bata A Sun W Ho C-P Vuppugalla R. Preclinical antitumor activity of a CC chemokine receptor (CCR) 2/5 dual antagonist as monotherapy and in combination with immune checkpoint blockade. Cancer Res. (2018) 78:3760–0. doi: 10.1158/1538-7445.AM2018-3760

[174] Lin KL Sweeney S Kang BD Ramsburg E Gunn MD. CCR2-antagonist prophylaxis reduces pulmonary immune pathology and markedly improves survival during influenza infection. J Immunol. (2011) 186:508–15. doi: 10.4049/jimmunol.1001002 PMC3723340 21098218

[175] de Zeeuw D Bekker P Henkel E Hasslacher C Gouni-Berthold I Mehling H. The effect of CCR2 inhibitor CCX140-B on residual albuminuria in patients with type 2 diabetes and nephropathy: a randomised trial. Lancet Diabetes Endocrinol. (2015) 3:687–96. doi: 10.1016/S2213-8587(15)00261-2 26268910

[176] Pienta KJ Machiels J-P Schrijvers D Alekseev B Shkolnik M Crabb SJ. Phase 2 study of carlumab (CNTO 888), a human monoclonal antibody against CC-chemokine ligand 2 (CCL2), in metastatic castration-resistant prostate cancer. Investigational New Drugs. (2013) 31:760–8. doi: 10.1007/s10637-012-9869-8 22907596

[177] Loberg RD Ying C Craig M Yan L Snyder LA Pienta KJ. CCL2 as an important mediator of prostate cancer growth in vivo through the regulation of macrophage infiltration. Neoplasia. (2007) 9:556–62. doi: 10.1593/neo.07307 PMC1939930 17710158

[178] Sandhu S Fong P Frentzas S Patnaik A Papadopoulos K Tromp B. First-in-human, first-in-class, phase I study of a human monoclonal antibody CNTO 888 to the CC-chemokine ligand 2 (CCL2/MCP-1) in patients with solid tumors. J Clin Oncol. (2009) 27:e13500–0. doi: 10.1200/jco.2009.27.15_suppl.e13500

[179] Fetterly GJ Aras U Meholick PD Takimoto C Seetharam S McIntosh T. Utilizing pharmacokinetics/pharmacodynamics modeling to simultaneously examine free CCL2, total CCL2 and carlumab (CNTO 888) concentration time data. J Clin Pharmacol. (2013) 53:1020–7. doi: 10.1002/jcph.140 23878055

[180] Raghu G Martinez FJ Brown KK Costabel U Cottin V Wells AU. CC-chemokine ligand 2 inhibition in idiopathic pulmonary fibrosis: a phase 2 trial of carlumab. Eur Respir J. (2015) 46:1740–50. doi: 10.1183/13993003.01558-2014 26493793

[181] Dudal S Subramanian K Flandre T Law W Lowe P Skerjanec A. Integrated pharmacokinetic, pharmacodynamic and immunogenicity profiling of an anti-CCL21 monoclonal antibody in cynomolgus monkeys. MAbs. (2015). doi: 10.1080/19420862.2015.1060384 PMC4622749 26230385

[182] Davda JP Hansen RJ. Properties of a general PK/PD model of antibody-ligand interactions for therapeutic antibodies that bind to soluble endogenous targets. MAbs. (2010). doi: 10.4161/mabs.2.5.12833 PMC2958579 20676036

[183] Bonapace L Coissieux M-M Wyckoff J Mertz KD Varga Z Junt T. Cessation of CCL2 inhibition accelerates breast cancer metastasis by promoting angiogenesis. Nature. (2014) 515:130–3. doi: 10.1038/nature13862 25337873

[184] Keklikoglou I De Palma M. Metastasis risk after anti-macrophage therapy. Nature. (2014) 515:46–7. doi: 10.1038/nature13931 25337881

[185] Mora E Guglielmotti A Biondi G Sassone-Corsi P. Bindarit: an anti-inflammatory small molecule that modulates the NFκB pathway. Cell Cycle. (2012) 11:159–69. doi: 10.4161/cc.11.1.18559 PMC3356824 22189654

[186] Mirolo M Fabbri M Sironi M Vecchi A Guglielmotti A Mangano G. Impact of the anti-inflammatory agent bindarit on the chemokinome: selective inhibition of the monocyte chemotactic proteins. Eur Cytokine Netw. (2008) 19:119–22. 10.1684/ecn.2008.0133 18775807

[187] Shen Z Kuang S Zhang M Huang X Chen J Guan M. Inhibition of CCL2 by bindarit alleviates diabetes-associated periodontitis by suppressing inflammatory monocyte infiltration and altering macrophage properties. Cell Mol Immunol. (2021) 18:2224–35. doi: 10.1038/s41423-020-0500-1 PMC8429574 32678310

[188] Ble A Mosca M Loreto Di G Guglielmotti A Biondi G Bombardieri S. Antiproteinuric effect of chemokine C-C motif ligand 2 inhibition in subjects with acute proliferative lupus nephritis. Am J Nephrol. (2011) 34:367–72. doi: 10.1159/000330685 21876349

[189] Severini C Passeri PP Ciotti M Florenzano F Possenti R Zona C. Bindarit, inhibitor of CCL2 synthesis, protects neurons against amyloid-β-induced toxicity. J Alzheimer's Dis. (2014) 38:281–93. 10.3233/JAD-131070 23948942

[190] Ge S Shrestha B Paul D Keating C Cone R Guglielmotti A. The CCL2 synthesis inhibitor bindarit targets cells of the neurovascular unit, and suppresses experimental autoimmune encephalomyelitis. J Neuroinflamm. (2012) 9:171. doi: 10.1186/1742-2094-9-171 PMC3488971 22788993

[191] Huang S-Y Chang S-F Liao K-F Chiu S-C. Tanshinone IIA inhibits epithelial-mesenchymal transition in bladder cancer cells via modulation of STAT3-CCL2 signaling. Int J Mol Sci. (2017) 18:1616. doi: 10.3390/ijms18081616 28757590 PMC5578008

[192] Chen C-Y Su C-M Huang Y-L Tsai C-H Fuh L-J Tang C-H. CCN1 induces oncostatin M production in osteoblasts via integrin-dependent signal pathways. PloS One. (2014) 9:e106632. doi: 10.1371/journal.pone.0106632 25187949 PMC4154729

[193] Du X Liu ZY Tao XX Mei YI Zhou DQ Cheng K. Research progress on the pathogenesis of knee osteoarthritis. Orthopaedic Surg. (2023) 15:2213–24. doi: 10.1111/os.13809 PMC10475681 37435789

[194] Xia L Lu J Xiao W. Blockage of TNF-α by infliximab reduces CCL2 and CCR2 levels in patients with rheumatoid arthritis. J Invest Med. (2011) 59:961–3. doi: 10.2310/JIM.0b013e31821c0242 21527853

[195] Lin YC Lin YC Huang MY Kuo PL Wu CC Lee MS. Tumor necrosis factor-alpha inhibitors suppress CCL2 chemokine in monocytes via epigenetic modification. Mol Immunol. (2017) 83:82–91. doi: 10.1016/j.molimm.2017.01.009 28113136

[196] Xu L Zhang Y Wang Q Zhao J Liu M Guo M. Bi-specific antibodies with high antigen-binding affinity identified by flow cytometry. Int Immunopharmacol. (2015) 24:463–73. doi: 10.1016/j.intimp.2014.12.011 25526913

[197] Zhao Q. Bispecific antibodies for autoimmune and inflammatory diseases: clinical progress to date. BioDrugs. (2020) 34:111–9. doi: 10.1007/s40259-019-00400-2 31916225

[198] Fleischmann RM Bliddal H Blanco FJ Schnitzer TJ Peterfy C Chen S. A phase II trial of lutikizumab, an anti–interleukin‐1α/β dual variable domain immunoglobulin, in knee osteoarthritis patients with synovitis. Arthritis Rheumatol. (2019) 71:1056–69. doi: 10.1002/art.40840 30653843

[199] Lacy SE Wu C Ambrosi DJ Hsieh C-M Bose S Miller R. Generation and characterization of ABT-981, a dual variable domain immunoglobulin (DVD-IgTM) molecule that specifically and potently neutralizes both IL-1α and IL-1β. MAbs. (2015). doi: 10.1080/19420862.2015.1026501 PMC4622731 25764208

[200] Fischer J Hueber AJ Wilson S Galm M Baum W Kitson C. Combined inhibition of TNFα and IL-17 as therapeutic opportunity for treatment in rheumatoid arthritis: Development and characterization of a novel bispecific antibody. Arthritis Rheum. (2015) 67:51–62. doi: 10.1002/art.38896 25303306

[201] Adnan ML. IL-17/TNF-α BISPECIFIC ANTIBODIES AS NEW THERAPEUTIC APPROACH TO RHEUMATOID ARTHRITIS. Turkish Med Student J. (2020) 7:37–40. doi: 10.4274/tmsj

[202] Singh R Lillard JW Jr. Nanoparticle-based targeted drug delivery. Exp Mol Pathol. (2009) 86:215–23. doi: 10.1016/j.yexmp.2008.12.004 PMC3249419 19186176

[203] Kang L-J Yoon J Rho JG Han HS Lee S Oh YS. Self-assembled hyaluronic acid nanoparticles for osteoarthritis treatment. Biomaterials. (2021) 275:120967. doi: 10.1016/j.biomaterials.2021.120967 34153786

[204] Jin G-Z. Current nanoparticle-based technologies for osteoarthritis therapy. Nanomaterials. (2020) 10:2368. doi: 10.3390/nano10122368 33260493 PMC7760945

[205] Mei X Villamagna IJ Nguyen T Beier F Appleton CT Gillies ER. Polymer particles for the intra-articular delivery of drugs to treat osteoarthritis. Biomed Materials. (2021) 16:042006. doi: 10.1088/1748-605X/abee62 33711838

